# Newly Characterized Porcine Epidemic Diarrhea Virus GII Subtype Strain

**DOI:** 10.1155/2023/5544724

**Published:** 2023-05-09

**Authors:** Jiarong Yu, Pengfei Chen, Ruilin Liu, Mengqin Lao, Junrui Zhu, Shuting Zhou, Jijie Jiang, Shijing Huang, Wu Tong, Yifeng Jiang, Fei Gao, Lingxue Yu, Hai Yu, Changlong Liu, Zhibiao Yang, Guangzhi Tong, Yanjun Zhou

**Affiliations:** ^1^Shanghai Veterinary Research Institute, Chinese Academy of Agricultural Sciences, Shanghai 200241, China; ^2^Institute of Animal Science and Veterinary Medicine, Shanghai Academy of Agricultural Science, Shanghai, China; ^3^Shanghai Key Laboratory of Veterinary Biotechnology, School of Agriculture and Biology, Shanghai Jiaotong University, Shanghai 200240, China; ^4^Jiangsu Co-Innovation Center for Prevention and Control of Important Animal Infectious Diseases and Zoonoses, Yangzhou University, Yangzhou 225009, China

## Abstract

Diarrhea outbreaks in piglets on pig farms are commonly attributed to porcine epidemic diarrhea virus (PEDV) infection. This research analyzed the S gene prevalence variation and recombination patterns in PEDV GII strains. Throughout the previous two years, 172 clinical samples of piglet diarrhea have been collected, from which 24 PEDV isolates have been isolated. Analysis of the evolutionary relationships among all 24 S genes revealed that 21 were most closely related to strains within the GII-a subgroup. The 2 isolates grouped into one clade with the GII-b subgroup. According to the mutation analysis of the amino acids (aa) that encode the S protein, 43 of the common aa in strains of the GII subtype were found to have undergone a change in polarity or charge, and 36 of these aa had a mutation frequency of more than 90%. Three different aa mutation sites were identified as exclusive to GII-a subtype strains. The genomes of three PEDV isolates were sequenced, and the resulting range in genome length was 28,035−28,041 nt. The results of recombination analysis showed that the SD1 isolate is a novel strain recombinant from the foreign S-INDEL strain and a domestic GII subtype strain. Based on the findings, the PEDV GII-a strain has been the most circulating strain in several parts of China during the previous two years. Our study reveals for the first time the unique change of aa mutations in the S protein of the GII-a subtype strain and the new characteristics of the recombination of foreign strains and domestic GII subtype strains, indicating that it is crucial to monitor the epidemic dynamics of PEDV promptly to prevent and control the occurrence of PED effectively.

## 1. Introduction

Acute watery diarrhea, vomiting, dehydration, and high mortality in piglets are hallmarks of porcine epidemic diarrhea (PED), an intestinal illness of pigs that can have a fatality rate as high as one hundred percent in newborn piglets [1–3]. Transmission of PED was rare in China and elsewhere between 1978 and 2010 [4, 5]. Commercial CV777 vaccinations were used extensively, and the PED outbreak in Asia was eventually contained. Nevertheless, a new pandemic strain of porcine epidemic diarrhea virus (PEDV) with a mutated genome emerged in China towards the end of 2010. When the original CV777 commercial vaccination failed to protect piglets from infection with the altered PEDV strain, a massive PED outbreak ensued, resulting in the deaths of millions of pigs and incurring significant economic losses for China's pig sector [6–8]. In 2013, the United States reported the first mutant strain of PEDV, and within a few months, the outbreak rapidly spread to 33 states and over 8,200 pig farms, resulting in the loss of nearly 7 million piglets [9]. Similar PED epidemics were subsequently detected in South Korea, Japan, and Taiwan, gaining global attention [10–12]. Up to now, there are still reports of PED throughout Asia, Europe, and North America where pigs are raised. The epidemic and variability of PED have severely jeopardized the growth of the global pig industry. PEDV is a single-stranded positive-sense RNA virus with an envelope that is a member of the family Coronaviridae and the genus *Alpha coronaviru*s [13]. PEDV contains seven open reading frames (ORFs): replicase protein polymer proteins (ppla and pplab), spike protein (S), ORF3 protein, small membrane protein (E), membrane glycoprotein (M), and nucleocapsid protein (N) [14, 15]. Presently, PEDV prevalence worldwide is divided into two genotypes: the GI subtype represented by CV777 and the GII subtype represented by the mutant PEDV strain. GII subtypes are divided into two subgroups: GII-a and GII-b. GII-a is an emerging strain. Its main feature is the recombination in the region of the S gene, resulting in a higher virulence of the emerging recombined strain than the GI subtype strain. Representative strains mainly include AH2012 [16], ZMDZY [17], and PC21A [18]. In addition to the previous subtypes of strains, there are also related reports on S-INDEL, and it is also reported to be classified as a new subtype GII-c, representing that the strains mainly include: The Korean strain KNU-1406-1 [19] in Asia, the German strain L00721/GER [20], the French strain FR/001 [21] in Europe, and in America, the American strain OH851 [22]. The S gene can be an important reference gene for PEDV typing.

Mutations in the coronavirus S gene can alter the efficiency of the virus binding to receptors and the immunogenicity, resulting in new, more transmissible, and pathogenic strains. In the alpha coronavirus genus, the mutation of the M1058L site of feline coronavirus (FCoV) leads to changes in the cell tropism of the virus, and the virus initiates systemic infection of the host through the intestinal system, while the I507L site of human coronavirus (HCoV-NL63) improves the ability of viruses to enter host cells [23, 24]. Among beta coronaviruses, the mutations at the D614G and N501Y sites of SARS-CoV-2 enhanced the infectivity and transmission of the virus, while the mutation of the P681R increased the virus' membrane fusion and pathogenicity [25–27]. Similar to the function of the S protein of other coronaviruses, the S protein of PEDV is mainly responsible for inducing neutralizing antibodies, specifically binding to host cell receptors, and mediating cell membrane fusion [28–30]. The mutation, insertion, and deletion of the S gene may be related to the virus' pathogenicity and tissue tropism. Previous studies have shown that the N-terminal region (NTD) of the PEDV S gene has a 197 aa deletion, which reduces the pathogenicity of the strain in piglets [31, 32]. The absence of 204-aa in the 713–916 site in the S protein of the Korean strain (MF3809/2008) altered the tissue tropism of the strain [11]. Multiple recombination events were identified in the S gene, demonstrating that alterations in the PEDV S gene are directly related to the genetic variety of PEDV strains [33]. This study tracked the prevalence of PEDV in pig farms experiencing piglet diarrhea outbreaks from 2020 to 2021 and used the sequencing results of the PEDV genome to conduct an in-depth analysis of the mutation characteristics of the S gene to understand better the epidemic dynamics and genome mutation characteristics of the currently circulating PEDV. It provides a reference for predicting the transmission capacity and infectivity of new PEDV strains.

## 2. Materials and Methods

### 2.1. Collection and Preparation of Clinical Samples

From 2020 to 2021, 172 clinical samples, including intestinal tissue and contents, were collected from different pig farms with piglet diarrhea in Zhejiang, Fujian, Shanghai, Jiangsu, and Shandong (Supplementary Table 1). The intestinal contents were washed with PBS and centrifuged at 15,000*g* at 4°C, and the supernatant was collected; 0.5 g of the intestinal tissue was removed, and 1 ml of sterile PBS was added, ground into tissue homogenate, centrifuged at 15,000*g* at 4°C, the supernatant was collected, and it was stored at −80°C for future use.

### 2.2. Sample Detection

The RNA in the sample treatment solution was extracted according to the RNA extraction instructions (E.Z.N.A Total RNA Kit), and the sample cDNA was produced using the RevertAid First Strand cDNA Synthesis Kit. Using the porcine enteric coronavirus and PEDV FJzz1 (GenBank accession no. MK288006.1) conservative N gene detection primers for specific PCR amplification as reported earlier [34]. The reaction system consisted of ddH_2_O 7 *μ*l, upstream primer 1 *μ*l, downstream primer 1 *μ*l, cDNA 1 *μ*l, 2 × LA Taq Premix 10 *μ*l, and a total volume of 20 *μ*l; the reaction protocol consisted of predenaturation (98°C, 30 s), denaturation (98°C, 10 s), annealing (57°C, 30 s), extension (72°C, 1 min), 35 cycles, and final extension (72°C, 10 min). The results were observed by 1% agarose gel electrophoresis.

### 2.3. S Gene and PEDV Full-Length Gene Amplification and Sequence Analysis

As indicated previously, positive samples were amplified using three pairs of primers that amplified PEDV S by overlapping and spanning the complete S gene. LA Taq was used to amplify 11 overlapping fragments that spanned the whole viral genome sequence for the cloning of the full-length genome. The amplification conditions were: predenaturation at 94°C for 3 min, denaturation at 94°C for 30 s, 49.8°C ∼Anneal at 59.0°C for 30 s, extension at 72°C for 6 min, and extension at 72°C for 10 min after 32 cycles. Amplification system: 0.5 *µ*L of LA Taq (5 U/*µ*L), 5 *µ*L of 10 × LA PCR buffer II (Mg^2+^ Plus), 8 *µ*L of dNTP mixture (2.5 mM each), 1 *µ*L of cDNA, 1 *µ*L of upstream and downstream primers, and 33.5 *µ*L of RNA-free water. The article's isolated strains have been submitted to the NCBI database (OQ349200–OQ349226). The amplification of PEDV N, S, and genome were mainly carried out using specific primers for N and S genes (Supplementary Tables 2 and 3). After recovering the PCR products from the gel, they were cloned into the pMD18-T vector and then sequenced by Sanger. The sequence analysis of 24 S genes and 3 full gene sequences was performed using the EditSeq tool in DNASTAR, while the multiple sequence alignment tool of MAFFT 7 [35] was utilized for multiple sequence alignment and the DNAMAN 6.0 software (Lynnon BioSoft) was exploited for protein sequence alignment.

### 2.4. Phylogenetic Analysis and S Gene Mutation Analysis

Based on the findings given in the publications by Guo et al., we reviewed the findings and initially categorized the representative strains of PEDV by country and subtype [36–38]. After this, we obtained all 743 PEDV full-length gene sequences published on the NCBI website between 1978 and 2020, and removed strains with incomplete gene sequences. Analyses of homology were conducted between supplementary and typical strains. The complementary strains were then subjected to homology analysis with representative strains, and one of the complementary strains with a similarity higher than 99.00% was chosen for subsequent analysis together with the reference strain. Finally, we selected the complete genome sequences of 422 strains of PEDV (*n* = 422). The selected sequences were then subjected to multiple sequence alignments using MAFFT 7 [35]. Similarly, we chose 86 complete S gene reference strain sequences with the preceding guidelines. The reference strain CV777 and the variant strain AJ1102 were selected as the representative strain of GI and GII genotype, respectively (Supplementary Tables 4 and 5). The full-length genome sequence and complete S gene obtained in this study used MEGA-7 software to create a phylogenetic tree of neighboring relationships, which was then annotated using Chiplot software (https://www.chiplot.online/). The obtained S gene sequence was aligned by MAFFT 7, and the CV777 strain (GenBank accession no. AF353511.1) served as the reference strain. The spike aa mutation was analyzed using BioAider V1.334.

### 2.5. Molecular Modeling Analysis of PEDV S Protein

After aligning the aa sequences of the PEDV S protein, the online software SWISS-MODEL (https://swissmodel.expasy.org/) was chosen to model the PEDV S region's tertiary structure. CV777, a representative strain of the GI subgroup, was used for molecular modeling. Simultaneously, PEDV-LYG (GenBank: KM609212.1) and AJ1102 (GenBank accession no. JX188454.1) were selected as the representative strain of GII-a subtype and GII-b subtype, respectively, so as to analyze the difference of S protein models between the GI and GII subgroup. The monomeric tertiary structure of S was generated and studied using the molecular viewer Pymol (version 3.7.6). The differences of the S protein between the GI and GII subtype strains were analyzed by using model comparisons.

### 2.6. Recombination Analysis

The obtained full-length PEDV gene sequence was analyzed using RDP4, and seven methods were used to identify recombination events [39]. The detected recombination events were further investigated using Bootscan in SimPlot v3.5 [40] with a window size of 500 bp and a step size of 20 bp. The eventual recombination was determined by the findings provided by the SimPlot program. The sites of recombinant parental strains and breakpoints were found using SimPlot findings.

## 3. Results

### 3.1. Phylogenetic Analysis of PEDV S Gene

**Figure 1:**
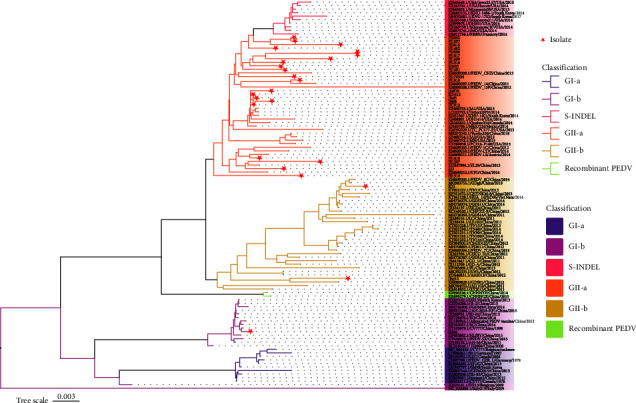
Phylogenetic analysis based on the full-length PEDV S gene. The red star represents the 24 strains isolated in the laboratory.

This study discovered 39 PEDV-positive samples out of 172 clinical samples of piglet diarrhea, with a positive rate of 22.67%. Among the positive samples, 24 complete PEDV S genes were obtained. Compared to CV777 (GenBank accession no. AF353511.1), the S genes of the 24 isolates have nucleotide sequence homology of 91.48–96.87% and aa homology of 88.21–94.67%. Compared to AJ1102 (GenBank accession no. JX188454.4), the nucleotide and aa sequence identities range from 93.35–98.58% to 90.51–97.41%, respectively. The result shows that the 24 strains of S genes obtained in this study are closely related to GII. The homology with the recently popular mutant strain of PEDV is high. The S gene phylogenetic tree was constructed using the 24 PEDV S gene sequences and 86 reference strains from GenBank obtained in this investigation. The results of this study indicate that all strains can be divided into two genotypes, GI and GII, with the SHxn strain belonging to the GI-b subgroup, SD1 and JSyj10 to the GII-b subgroup, and the remaining 21 strains to the GII-a subgroup, with 87.50% of the strains belonging to the GII-a subgroup (Figure 1). It is suggested that GII-a subgroup PEDV strains have become the predominant strains in some regions of our nation, and it is important to monitor the epidemic dynamics of these strains time-dependent.

### 3.2. Analysis of S Gene Mutation of PEDV in China

To study the features of amino acid mutations in PEDV S genes of various gene subtypes, we downloaded from GenBank 125 S gene sequences discovered in China after 2010. Utilizing CV777 as a reference strain, the S genes of 125 emerging strains and 24 strains collected in this investigation were analyzed for mutations. In GII subgroup strains, 46 aa mutations in GII-a lead to changes in the polarity or charge of aa; 43 aa mutations in GII-b lead to changes in the polarity or charge of aa. There were 43 common amino acid mutations in the GII subtype strains, and the mutation frequency of 36 common aa exceeded 90.00%. Moreover, among these consensus amino acid mutations, 34 mutated aa were located in the N-terminal domain (NTD), 3 mutated aa were located in the C-terminal domain (CTD) (I360T, E369Q, and S458A), 2 mutated aa were located in the SD region (A609E and N711D), and 4 mutated aa were located in S2 (S1048A, G1177D, S1236R, and R1306Q) (Table 1). We found 3 unique aa mutation sites in the GII-a subgroup, ^139^N/D, ^531^A/S, and ^979^A/S. We speculate that the previous mutation sites may be used to classify the PEDV strains gene. To test this hypothesis, we collected 290 PEDV complete S gene sequences of the GII-a subtype from NCBI (Supplementary Table 6) and analyzed the previous mutation sites. The results showed that for the 46 aa mutations of GII-a, the site mutation frequency exceeded 90.00%, and GII-a subgroup strains with ^139^N/D-^531^S-^979^S mutation pattern accounted for 97.93% (Table 2). These three unique mutation patterns are relatively conserved in GII-a subgroup strains, which may be used as a reference for the additional classification of PEDV GII subgroup strains.

### 3.3. Molecular Modeling of GII Subtype S Gene

**Figure 2:**
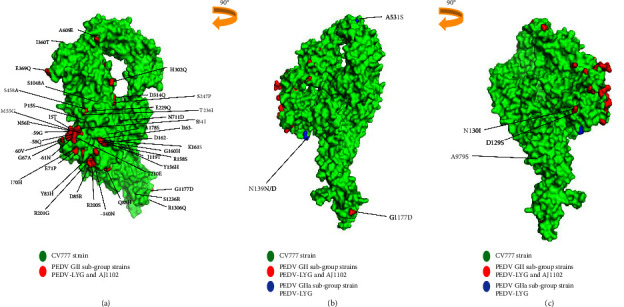
Comparative analysis of S protein modeling of PEDV CV777, PEDV-LYG strains, and AJ1102. The red in (a) indicates the sites with a high frequency of aa mutations in the S protein structure of the PEDV-LYG strains and AJ1102, and the green indicates the 3D structure of the S protein of the CV777 strain; (b) shows the structure of (a) rotated 90° clockwise, the blue in the figure indicates the unique mutation of the S protein of the GII-a subtype strain PEDV-LYG. Red indicates the sites with a high frequency of aa mutation in the S protein structure of the GII subtype strains PEDV-LYG and AJ1102, and green indicates the 3D structure of the S protein of the CV777 strain; (c) is the structural diagram of (b) rotated 90° clockwise, GII-a subtype strain PEDV-LYG blue the A979S site is partially buried inside.

**Figure 3:**
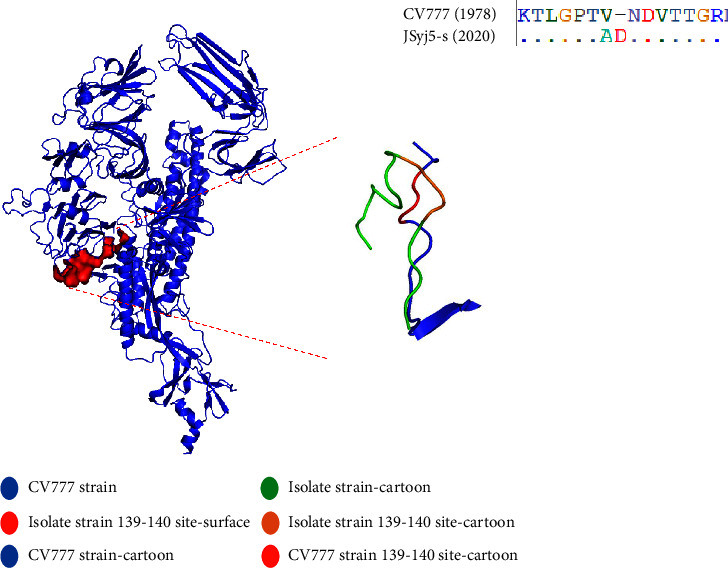
Comparative analysis of the S protein model between the PEDV CV777 strain and the laboratory-isolated JSyj5 strain. The cartoon displayed by PEDV CV777 is represented by blue, the cartoon of the isolated strain JSyj5 is represented by green, the mutant surface of the isolated strain JSyj5 is represented by red, the cartoon of the mutation point of PEDV CV777 is represented by red, and the mutation point of the isolated strain JSyj5 is represented by red. The cartoon is represented by orange.

To illustrate the distribution of the 46 aa mutations of the GII subtype on the spike protein, we modeled and analyzed the GII subtype reference strain (PEDV-LYG and AJ1102) and GI subtype reference strain (CV777) S protein. The results showed that, except for I119T, A178S, S458A, A979S, and S1048A, the remaining 41 mutations of aa are located on the surface of the S protein (Figure 2). However, the 3 unique mutants aa of the GII-a strain are respectively in the random coil region or the *β*-sheet region. The unique aa mutation ^139^N/D in the NTD region changes the polarity of the aa, resulting in a significant shift in the random coil (Figure 3). In contrast, the other two unique aa mutations did not cause significant changes in the structure of the corresponding positions.

### 3.4. Phylogenetic Analysis of PEDV Whole Gene Sequence

**Figure 4:**
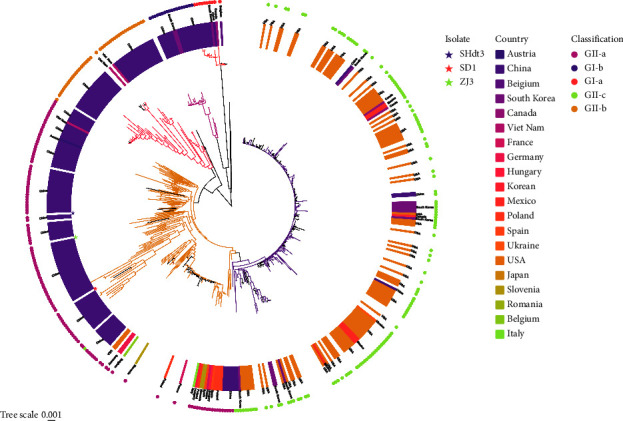
Genotyping of global PEDV and isolated strains based on full-length genomic sequence analyses. Stars represent isolated laboratory strains, branch color represents strain classification, and squares represent different countries.

**Figure 5:**
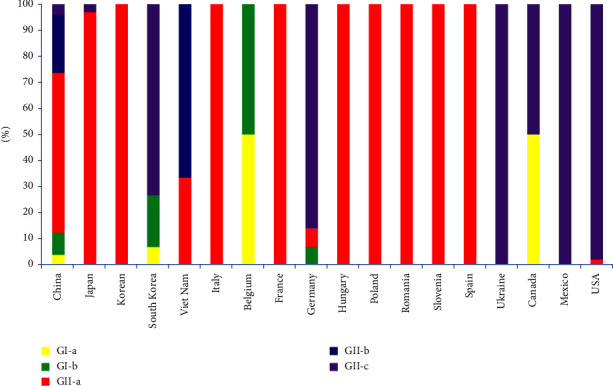
The prevalence of different subtypes of PEDV in various countries in the world. Yellow indicates GI-a, green indicates GI-b, red indicates GII-a, blue indicates GII-b, and purple indicates GII-c.

To better comprehend the genetic evolution characteristics of the currently prevalent PEDV, we selected three PEDV isolates SD1 (GenBank accession no. OQ349200), SHdt3 (GenBank accession no. OQ349201), and ZJ3 (GenBank accession no. OQ349202) for full-length genome sequencing. The sequencing results showed that the genome length of the SD1 strain was 28,035 bp, whereas that of the SHdt3 strain was 28,038 bp. The ZJ3 strain genome is 28,041 bp (excluding the poly (A) tail). Further homology analysis was carried out between them and three typical reference strains: CV777 (GenBank: AF353511.1), AJ1102 (GenBank: JX188454.4), and OH851 (GenBank accession no. KJ399978.1). The results showed that the genetic relationship with the representative strain AJ1102 of the GII genotype in the three strains is relatively close, and its nucleotide homology is 98.25–98.76%, and the aa homology is 96.45–97.89% (Table 3). We constructed the phylogenetic tree based on PEDV whole genome sequences of the 425 strains including 422 reference strains collected from NCBI and three PEDV strains isolated in this study using the neighbor-joining method. The results indicated 392 strains with GII genotype, accounting for 92.24% (392/425). These GII genotype isolates were divided into three subgroups, GII-a, GII-b, and GII-c, and the SD1, ZJ3, and SHdt3 isolates obtained in this study all belonged to the GII-a subgroup (Figure 4). The statistical results show that among the 425 PEDV strains, 243 strains in Asia, the GII-a subgroup strains accounted for 62.96% (153/243) and the GII-c subgroup strains accounted for 7.82% (19/243); among the 49 strains in Europe, GII-a accounted for 38.78% (19/49) and GII-c accounted for 53.06% (26/49); and among the 128 strains in America, GII-a accounted for 1.56% (2/128) of these, while GII-c accounted for 96.88% (124/128) (Figure 5, Table 4). The previous study demonstrates that the GII subtype remains the most prevalent. In addition, the GII-a subgroup strains dominate in Asia, and the GII-c subgroup strains dominate in Europe and America.

### 3.5. Recombination Analysis of Isolated Virus Strains

**Figure 6:**
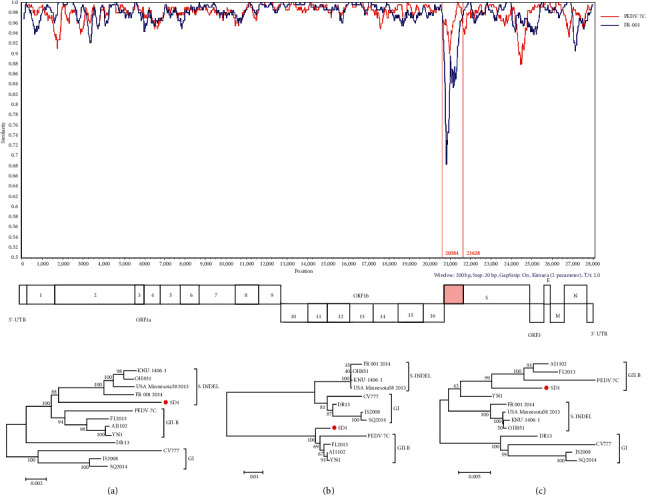
Gene recombination analysis of SD1 isolate. In (a) the red color represents the GII-b strain PEDV-7C and the blue color represents the S-INDEL strain FR 001; in (b) the circle represents the isolated strain SD1.

Recombination is the primary mechanism through which viral genetic variation evolves. To analyze the potential recombination events of the three PEDV strains SD1, ZJ3, and SHdt3 isolated in this study, 12 representative PEDV strains were selected from 422 reference PEDV strains for a recombinant analysis together with the three isolates (Supplementary Table 7). The results showed that 6 of the 7 detection methods of RDP4 supported potential recombination events in SD1. However, SHdt3 and ZJ3 did not detect recombination events. Among them, the SD1 strain uses the FR 001 strain as the parent virus backbone, the S section of PEDV-7C (secondary parent strain) is chimerized in the nt 20,584−21,682 region, and the recombination region includes the NTD region of the S gene (Figure 6(a)). SimPlot software was used to compare the similarity between the recombinant and the reference strain. The results showed that the recombination breakpoints of the SD1 strain separated the regions into A (nt 1–20,583), B (nt 20,584−21,682), and C (nt 21,683−28,000). The results of phylogenetic evolution analysis showed that region A had the closest genetic relationship with the S-INDEL subgroup strain FR 001 and belonged to the same branch. B and C had the closest genetic relationship with GII-b subgroup strain PEDV-7C, which are in the same branch, indicating that the SD1 strain isolated in this study is a new strain generated by the recombination of S-INDEL-like strains and GII-b subgroup strains (Figure 6(b)).

## 4. Discussion

In recent years, PED has been the most prevalent and devastating enteric disease in the swine industry [5]. The continuous mutation of PEDV leads to increased PEDV genetic diversity, resulting in the inability of existing vaccines to achieve complete piglets protection, which causes a major challenge to the prevention and control of PED. Since PED was first reported in the UK in 1977, the prevalence of PEDV strains of the GI subtype has been dominant in countries worldwide [41]. At the end of 2010, a new highly pathogenic variant GII subtype PEDV strain was first discovered in China, triggering a large-scale PED outbreak [7, 8, 34]. Compared with classic strains such as CV777, the new mutant strain is characterized by four hypervariable regions in the S gene, among which the S1 region located in the V2 region is the region with the most obvious variation [42]. The insertion of nt 162, nt 170–180, and nt 413–415 and the deletion of nt 470–475 led to mutations in the neutralizing epitopes of the mutant strains, resulting in increased virulence of the GII subgroup strains and poor protective effect of the GI subgroup commercial vaccines [4, 43]. In recent years, to adapt to vaccine immunity and environmental pressure, PEDV has been continuously evolving.

**Figure 7:**
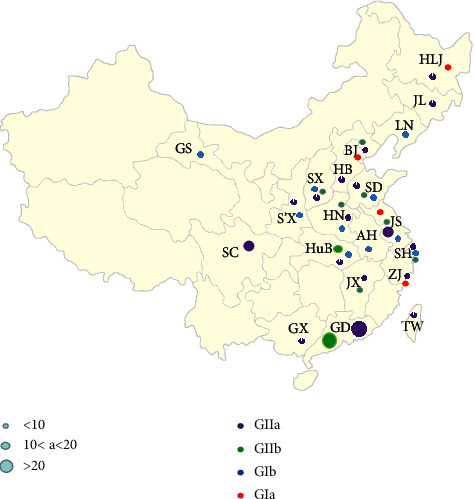
Geographical distribution of different subtypes of PEDV in various provinces of China. Different colors represent different subtypes, and the large and small circles represent the number of PEDVs. The information on the 21 provinces in China is as follows. HLJ, Heilongjiang; JL, Jilin; LN, Liaoning; BJ, Beijing; HB, Hebei; SD, Shandong; JS, Jiangsu; Anhui; JX, Jiangxi; S'X, Shanxxi; HuB, Hubei; SC, Sichuan; GS, Gansu.

This study confirmed that PEDV infection is still the major factor of piglet diarrhea from the clinical samples collected in the past two years. It is worth noting that the S gene phylogenetic analysis found that 21 strains isolated in this study belonged to the GII-a subgroup, two strains belonged to the GII-b subgroup (SD1 and JSyj10), and one strain (SHxn strain) belonged to the GI-b subgroup. Based on the results of phylogenetic analysis, we proposed that the present PEDV strains in parts of China are dominated by the GII-a subgroup strains, consistent with many previous reports [44, 45]. 188 PEDV S gene sequences were selected to investigate the prevalence of different PEDV genotypes and subgroups in China. The results show that about 60.11% (113/188) belonged to GII-a, 23.94% (45/188) belonged to GII-b, and 12.23% (23/188) belonged to the GI subtype. It was determined that the strains in China were mainly of the GII subtype, and the GII-a subgroup strains dominated the epidemic, which agrees with what has been found in other research [38]. At the same time, we conducted a statistical analysis of the 188 reported strains in our country. We found that the distribution of PEDV subtypes in the coastal areas of China is more complicated than that in the inland areas. One subtype is found in inland regions like Gansu (GI-b) and Sichuan (GII-a). However, multiple virus subtypes coexist in several coastal provinces (Figure 7). For example, Jiangsu Province has four subgroup strains: GI-a (4/188), GI-b (5/188), GII-a (11/188), and GII-b (2/188) all exist [46, 47], there are three subgroups of strains in Shandong, Henan, and other provinces and cities. Guangdong Province showed the highest number of GII subtype strains among the coastal cities, suggesting that it may be the transmission hub for early GII subtype viruses in China, spreading PEDV to other provinces. Compared to other provinces, Hubei Province has the second-most GII-b subtype strains, second only to Guangdong. In addition, being an interior city, Hubei Province has different subgroups coexisting. Thus, Hubei Province may serve as a viral transmission hub, allowing the virus to move from coastal to inland regions of our nation. According to the previous results, the coexistence of multi-subtype strains may be related to the pig trade between provinces, leading to diversifying the prevalence of PEDV subtypes.

Mutations in the S gene of coronaviruses can alter the binding effectiveness and immunogenicity of the S protein to receptors, resulting in strains that are more transmissible and infectious [25, 48, 49]. In SARS-CoV-2, deletions and mutations in the NTD and RBD regions of the S proteins interfere with antibody recognition and binding, thus contributing to the immune evasion of the virus [50, 51]. In the E3 (55−70aa), E4 (82−98aa), and E5 (126−141aa) regions of PEDV, neutralizing epitopes have been confirmed [52]. This study found that the GII-a subtype strains had two unique mutations (^139^N ⟶ ^139^D and ^531^A ⟶ ^531^S) in the NTD and CTD regions, and the 21 GII-a subtype isolates identified in this study also had these two mutations. The ^139^N ⟶ ^139^D in the NTD region causes the aa to change from uncharged to negatively charged, and then, the simulated structure was analyzed. It was found that the change in charge of this aa changed its random coil structure, and this structural change may affect the mutant strains. The adaptability of the virus helps the virus to evade the immune recognition of the host. However, ^531^A to ^531^S in the CTD region causes the aa properties to change from nonpolar to polar, making it mutate from hydrophobic to hydrophilic aa, which may relatively affect the antibody binding efficiency. The study's results suggest that the mutations of the specific aa in NTD and CTD may affect the immune evade and infection capabilities of PEDV variants. In addition, this may also be one of the important reasons why the prevalence of PEDV has gradually changed from the GII-b subtype to the GII-a subtype.

At the same time, it was found in this study that compared to CV777, there are 36 aa mutations in the GII subtype with a higher frequency (over 90.00%), of which 43 common aa mutations are mainly concentrated in the S1 gene NTD of the S protein. There are as many as 34 mutation sites in the NTD region. These mutations may cause changes in the conformation of S1-NTD, affecting the pathogenicity or cell tropism of PEDV [53]. Moreover, 41.86% (18/43) of the mutations at these sites altered both the polarity and charge of the aa at the same time, 16.28% (7/43) of the aa changed only the polarity, and 41.86% (18/43) of only the charge of the aa was changed (Supplementary Table 9). In addition to the positions with high mutation frequency mentioned previously, three unique aa mutations were also found in the GII-a subgroup strains, which also changed the aa properties (specifically: ^139^N ⟶ ^139^N/D in the NTD region, ^531^A ⟶ ^531^S in the CTD region, and ^979^A ⟶ ^979^S in the S2 region.), mainly the aa substitute from nonpolar to polar, and the change of aa polarity may affect the change of protein structure, especially the aa changes in the NTD and CTD regions, which will allow the virus to evade the recognition of immune antibodies and increase the binding affinity of the virus to the receptor [54, 55]. For SARS-CoV-2, the change in the electrostatic charge of the aa of the Omicron variety increased the virus's transmissibility, enhancing the disease's infectiousness [56]. This study found that 18 aa in the mutant aa of the GII subtype strains changed from uncharged to negatively charged. This shift in change resembles the outcomes described by Omicron. Therefore, we speculate that the GII subtype PEDV spread of the pandemic strain may be strongly associated with these mutations. In addition, when counting the GII subtype aa mutations, we found that there are 3 unique aa mutations in the GII-a subgroup strains, which is a relatively conservative aa mutation pattern, namely: ^139^N/D-^531^S-^979^S, after thorough comparison and verification of 290 GII-a subtype strains collected in the GenBank database, we found that 284 GII-a subgroup strains have this conserved aa mutation pattern, which can be used to distinguish an auxiliary typing method for the GII-a subgroup strains, which is used to determine the typing of GII-a strains when PED is prevalent.

In addition to gene mutation, viral recombination is also an important way of viral genetic evolution. Recombination can change the pathogenicity and transmission ability of the virus. Previously, there have been reports of PEDV recombinant strains [57, 58]. In this study, we used seven algorithms of RDP4 to perform recombination analysis on 15 PEDV genome sequences (including 12 reference sequences selected from 422 strains and 3 isolated sequences isolated in our laboratory) and detected potential recombination events in 1 isolate, and these putative recombination events were further confirmed by the phylogenetic analysis. SD1 is a new recombinant strain between the S-INDEL and GII-b subtype strains. According to the analysis, the French FR 001 strain provided the SD1 strain with its genetic backbone, while the PEDV-7C, as a secondary parent strain, contributed a partial gene fragment covering the NTD of the S protein. Research has demonstrated that the NTD region of the PEDV S gene is the essential area for binding to sialic acid. Replacing the NTD region of the S gene may alter the binding activity with sialic acid molecules, altering the cell tropism of SD1, and improving its infectiousness [59]. Previously, Fan identified that PEDV variants may have originated from recombination between the earliest Chinese G1 genome strain, JS-2004-2, and an Asian Korean pandemic strain [37]. Notably, the SD1 isolate is a new strain produced by recombining the French strain and the GII subtype Chinese native strain. As far as we know, this is the first to discover the origin of the French and Chinese native PEDV strains According to the reports of recombination of PEDV strains, we speculate that the emergence of this strain may be related to the introduction from abroad because the introduction increases the chance of recombination between foreign strains and Chinese native strains.

In general, the initial recombination events between emerging variants may initially occur randomly in immunocompromised pig herds. Pig introduction between provinces, cities, and regions and from abroad also creates opportunities for recombination between different subtypes of viruses. Subsequently, the emerging virus continued to improve its host adaptability through aa mutation in pigs, thus promoting the evolution of PEDV. The results of this study also show that the recombination methods of PEDV strains currently prevalent in our country are gradually showing a trend of diversification, suggesting that we should introduce them carefully and control the continuous introduction of non-native pathogens from the source. In conclusion, continuous monitoring of PEDV genome mutations is of great significance for understanding the genetic variation of PEDV and developing new PEDV vaccines, and it is also of great reference value for correcting changes in viral epitopes recognized by vaccines.

## Figures and Tables

**Table 1:** Comparison of aa mutations of Spike protein between GII-a and GII-b.

Spike regions		Position	Mutation (GII-a)	Percentage (GII-a) (%)	Mutation (GII-b)	Percentage (GII-b) (%)	New mutation
		5	I5T	94.81	I5T	98.70	
		15	P15S	97.40	P15S	97.40	
		54	S54I	87.01	S54I	89.61	
		55	M55G	98.70	M55G	97.40	
		56	N56E	98.70	N56E	97.40	
		58	-58Q	96.10	-58Q	97.40	
		59	-59G	96.10	-59G	98.70	
	NTD	60	-60V	90.91	-60V	98.70	
		61	-61N	94.81	-61N	98.70	
		67	G67A	96.10	G67A	96.10	
		70	I70H	98.70	I70H	98.70	
		71	E71P	96.10	E71P	97.40	
		83	Y83H	94.81	Y83H	98.70	
		85	D85R	98.70	D85R	97.40	
		88	Q88H	98.70	Q88H	97.40	
		119	I119T	98.70	I119T	98.70	
		129	D129S	85.71	D129S	94.81	
		130	N130I	94.81	N130I	88.31	
		139	N139D	97.40	N139D	—	N139D
		140	-140N	83.12	-140N	62.33	
S1		156	Y156H	96.10	Y156H	96.10	
		158	R158S	96.10	R158S	97.40	
		160	G160H	96.10	G160H	98.70	
		161	K161S	96.10	K161S	98.70	
		162	D162-	98.70	D162-	98.70	
		163	I163-	98.70	I163-	98.70	
		178	A178S	96.10	A178S	98.70	
		200	R200S	96.10	R200S	98.70	
		201	R201G	98.70	R201G	98.70	
		210	T210E	97.40	T210E	98.70	
		229	E229Q	96.10	E229Q	98.70	
		236	T236I	93.51	T236I	93.51	
		247	S247P	97.40	S247P	93.51	
		302	H302Q	92.21	H302Q	98.70	
		314	D314Q	96.10	D314Q	96.10	
	CTD	360	I360T	98.70	I360T	93.51	
		369	E369Q	98.70	E369Q	98.70	
		458	S458A	96.10	S458A	97.40	
		531	A531S	96.10	A531S	—	A531S
	SD	609	A609E	89.61	A609E	97.40	
		711	N711D	98.70	N711D	98.70	
	S2	979	A979S	76.62	A979S	—	A979S
		1048	S1048A	89.61	S1048A	97.40	
S2		1177	G1177D	93.51	G1177D	94.81	
		1236	S1236R	94.81	S1236R	96.10	
		1306	R1306Q	87.01	R1306Q	94.81	
			Total: 76		Total: 73		

**Table 2:** Statistical changes in mutation sites and aa properties of PEDV GII-a subtype strains.

Sites	Mutations	Change in properties of aa	Number of mutations	Percentages (%)
I5T	Yes	(Nonpolar, noncharge) to (polar, noncharge)	287	98.97
P15S	Yes	(Nonpolar, noncharge) to (polar, noncharge)	288	99.31
S54I	Yes	(Polar, noncharge) to (nonpolar, noncharge)	288	99.31
M55G	Yes	(Nonpolar, noncharge) to (polar, noncharge)	270	93.10
N56E	Yes	(None, none) to (polar, negative-charge)	281	96.90
-58Q	Yes	(None, none) to (polar, noncharge)	283	97.58
-59G	Yes	(None, none) to (polar, noncharge)	285	98.28
-60V	Yes	(None, none) to (polar, noncharge)	282	97.24
-61N	Yes	(None, none) to (polar, noncharge)	282	97.24
G67A	Yes	(Polar, noncharge) to (nonpolar, noncharge)	283	97.59
I70H	Yes	(Nonpolar, noncharge) to (polar, positive-charge)	287	98.97
E71P	Yes	(Polar, negative-charge) to (nonpolar, noncharge)	284	97.93
Y83H	Yes	(Polar, noncharge) to (polar, positive-charge)	285	98.28
D85R	Yes	(Polar, negative-charge) to (polar, positive-charge)	282	97.24
Q88H	Yes	(Polar, noncharge) to (polar, positive-charge)	284	97.93
I119T	Yes	(Nonpolar, noncharge) to (polar, noncharge)	287	98.97
D129S	Yes	(Nonpolar, noncharge) to (polar, positive-charge)	283	97.59
N130I	Yes	(Polar, noncharge) to (nonpolar, noncharge)	283	97.59
N139N/D	Yes	(None, none) to (polar, negative-charge)	285	98.28
-140N	Yes	(None, none) to (polar, noncharge)	287	98.97
Y156H	Yes	(Polar, noncharge) to (polar, positive-charge)	278	95.86
R158S	Yes	(Polar, positive-charge) to (polar, noncharge)	283	97.59
G160H	Yes	(Polar, noncharge) to (polar, positive-charge)	285	98.28
K161S	Yes	(Polar, positive-charge) to (polar, noncharge)	283	97.59
D162-	Yes	(Polar, negative-charge) to (none, none)	289	99.66
I163-	Yes	(Nonpolar, noncharge) to (none, none)	289	99.66
A178S	Yes	(Nonpolar, noncharge) to (polar, noncharge)	286	98.62
R200S	Yes	(Polar, positive-charge) to (polar, noncharge)	285	98.28
R201G	Yes	(Nonpolar, noncharge) to (polar, noncharge)	283	97.59
T210E	Yes	(Polar, positive-charge) to (polar, noncharge)	286	98.62
E229Q	Yes	(Polar, negative-charge) to (polar, noncharge)	285	98.28
T236I	Yes	(Polar, noncharge) to (nonpolar, noncharge)	282	97.24
S247P	Yes	(Polar, noncharge) to (nonpolar, noncharge)	285	98.28
H302Q	Yes	(Polar, positive-charge) to (polar, noncharge)	285	98.28
D314Q	Yes	(Polar, negative-charge) to (polar, noncharge)	286	98.62
I360T	Yes	(Nonpolar, noncharge) to (polar, noncharge)	288	99.31
E369Q	Yes	(Polar, negative-charge) to (polar, noncharge)	289	99.77
S458A	Yes	(Polar, noncharge) to (nonpolar, noncharge)	289	99.66
A531S	Yes	(Nonpolar, noncharge) to (polar, noncharge)	284	97.93
A609E	Yes	(Nonpolar, noncharge) to (polar, negative-charge)	272	93.79
N711D	Yes	(Polar, noncharge) to (polar, negative-charge)	287	99
A979S	Yes	(Nonpolar, noncharge) to (polar, noncharge)	290	100.00
S1048A	Yes	(Polar, noncharge) to (nonpolar, noncharge)	286	98.62
G1177D	Yes	(Polar, noncharge) to (polar, negative-charge)	288	99.31
S1236R	Yes	(Polar, noncharge) to (polar, positive-charge)	288	99.31
R1306Q	Yes	(Polar, positive-charge) to (polar, noncharge)	283	97.59

**Table 3:** Nucleotide and amino acid homology analysis of isolated strains.

	*CV777 (GenBank: AF353511.1)*		*AJ1102 (GenBank: JX188454.4)*		*OH851 (GenBank: KJ399978.1)*	
	Nt (%)	Aa (%)	Nt (%)	Aa (%)	Nt (%)	Aa (%)
SD1	96.49	93.18	98.25	96.45	97.74	95.58
ZJ3	96.60	93.53	98.56	97.33	98.40	97.42
SHdt3	96.62	93.53	98.76	97.89	98.48	97.51

**Table 4:** Statistics of different subtypes of PEDV strains in various countries.

	China	Japan	Korean	South Korea	Viet Nam	Italy	Belgium	France	Germany	Hungary	Poland	Romania	Slovenia	Spain	Ukraine	Canada	Mecixo	USA
GI-a	7	0	0	1	0	0	1	0	0	0	0	0	0	0	0	2	0	0
GI-b	16	0	0	3	0	0	1	0	2	0	0	0	0	0	0	0	0	0
GII-a	114	36	1	0	1	1	0	2	2	2	6	2	3	2	0	0	0	2
GII-b	42	0	0	0	2	0	0	0	0	0	0	0	0	0	0	0	0	0
GII-c	7	1	0	11	0	0	0	0	25	0	0	0	0	0	1	2	6	116
Total	186	37	1	15	3	1	2	2	29	2	6	2	3	2	1	4	6	118

## Data Availability

The original contributions presented in the study are included in the article/Supplementary Material. Further inquiries can be directed to the corresponding authors.

## Supplementary Materials

Supplementary Materials Supplementary Table 1: information about samples collected in this study. Supplementary Table 2: primer sequences for S and N gene amplification. Supplementary Table 3: primer sequences for PEDV genome amplification. Supplementary Table 4: 425 PEDV strains with whole genome sequences in this study. Supplementary Table 5: 86 PEDV reference strains with complete S gene sequences in this study. Supplementary Table 6: 290 PEDV strains of the GII-a subtype with the full-length S gene sequences in this study. Supplementary Table 7: 12 representative strains for recombinant analysis. Supplementary Table 8: analysis of polarity and charge changes of the mutant aa. Supplementary Table S1: 125 reference strains used for sequence alignment and 23 strains isolated in this study.
