# Supplementary material for: Newly Characterized Porcine Epidemic Diarrhea Virus GII Subtype Strain
Source: Transbound Emerg Dis. 2023 May 9;2023:5544724. doi: 10.1155/2023/5544724 (PMC12017209; doi:10.1155/2023/5544724)
Supplement: Supplementary Materials — Supplementary Table 1: information about samples collected in this study. Supplementary Table 2: primer sequences for S and N gene amplification. Supplementary Table 3: primer sequences for PEDV genome amplification. Supplementary Table 4: 425 PEDV strains with whole genome sequences in this study. Supplementary Table 5: 86 PEDV reference strains with complete S gene sequences in this study. Supplementary Table 6: 290 PEDV strains of the GII-a subtype with the full-length S gene sequences in this study. Supplementary Table 7: 12 representative strains for recombinant analysis. Supplementary Table 8: analysis of polarity and charge changes of the mutant aa. Supplementary Table S1: 125 reference strains used for sequence alignment and 23 strains isolated in this study. [file 5544724.f1.zip › Supplementary Table 1 (1).docx]

**Supplementary Table 1. Information about samples collected in this study.**

| Province | Number of farms | Number of samples |
| --- | --- | --- |
| Fujian | 3 | 75 |
| Shanghai | 2 | 39 |
| Shandong | 2 | 16 |
| Zhejiang | 1 | 5 |
| Jiangsu | 2 | 37 |
| Total | 10 | 172 |
