# Supplementary material for: Newly Characterized Porcine Epidemic Diarrhea Virus GII Subtype Strain
Source: Transbound Emerg Dis. 2023 May 9;2023:5544724. doi: 10.1155/2023/5544724 (PMC12017209; doi:10.1155/2023/5544724)
Supplement: Supplementary Materials — Supplementary Table 1: information about samples collected in this study. Supplementary Table 2: primer sequences for S and N gene amplification. Supplementary Table 3: primer sequences for PEDV genome amplification. Supplementary Table 4: 425 PEDV strains with whole genome sequences in this study. Supplementary Table 5: 86 PEDV reference strains with complete S gene sequences in this study. Supplementary Table 6: 290 PEDV strains of the GII-a subtype with the full-length S gene sequences in this study. Supplementary Table 7: 12 representative strains for recombinant analysis. Supplementary Table 8: analysis of polarity and charge changes of the mutant aa. Supplementary Table S1: 125 reference strains used for sequence alignment and 23 strains isolated in this study. [file 5544724.f1.zip › Supplementary Table 2 (2).docx]

**Supplementary Table 2. Primer sequences for S and N gene amplification.**

| Names of primer | Sequence 5′~3′ | Length |
| --- | --- | --- |
| S1-U | AAGTTACCTGATGGCATTATG | 2034 bp |
| S1-L | AATAGCCAAACCCATTGAC |  |
| S2-U | GCCATCTTTGCCATACCTCT | 2504 bp |
| S2-L | CATGCGTAAACAAGACTAAGC |  |
| S3-U | GCCCAAACCCTCACTAAGT | 2067 bp |
| S3-L | CACAACCGAATGCTATTGACA |  |
| NPU | CCGCTATAGGACTCGTACTGAGGGT | 609 bp |
| NPL | TTTTCGCCCTTGGGAATTCTCCTC |  |
