# Supplementary material for: Newly Characterized Porcine Epidemic Diarrhea Virus GII Subtype Strain
Source: Transbound Emerg Dis. 2023 May 9;2023:5544724. doi: 10.1155/2023/5544724 (PMC12017209; doi:10.1155/2023/5544724)
Supplement: Supplementary Materials — Supplementary Table 1: information about samples collected in this study. Supplementary Table 2: primer sequences for S and N gene amplification. Supplementary Table 3: primer sequences for PEDV genome amplification. Supplementary Table 4: 425 PEDV strains with whole genome sequences in this study. Supplementary Table 5: 86 PEDV reference strains with complete S gene sequences in this study. Supplementary Table 6: 290 PEDV strains of the GII-a subtype with the full-length S gene sequences in this study. Supplementary Table 7: 12 representative strains for recombinant analysis. Supplementary Table 8: analysis of polarity and charge changes of the mutant aa. Supplementary Table S1: 125 reference strains used for sequence alignment and 23 strains isolated in this study. [file 5544724.f1.zip › Supplementary Table 3 (2).docx]

**Supplementary Table 3. Primer sequences for PEDV genome amplification.**

| Names of primer | Sequence 5′~3′ | Length |
| --- | --- | --- |
| PED-1U | ACTTAAAAAGATTTTCTATCTACGGATA | 2348bp |
| PED-1L | GTTCATGGGCCGTAATG |  |
| PED-2U | TGCTGGCACTGGTGTTAAGTA | 2168bp |
| PED-2L | TCACCCTCGAAATCAAT |  |
| PED-3U | AGCTTGGCGTCGAGGATG | 2928bp |
| PED-3L | AATAAGAAACACACCGCAACA |  |
| PED-4U | CTTTGGCATCCGTACTATCC | 4906bp |
| PED-4L | CACTCACTAGCAAGGCAATTA |  |
| PED-5U | CGTAGCCGAACACGATTTC | 2336bp |
| PED-5L | TCATGCTTAGATAACGGGTAG |  |
| PED-6U | CGTCGTTGAGTATTATGGTTA | 5201bp |
| PED-6L | AGTGGCAACCAACGTCGTAAG |  |
| PED-7U | AAGTTACCTGATGGCATTATG | 2042bp |
| PED-7L | AGTAGCCAAACCCATTGAC |  |
| PED-8U | GCCATCTTTGCCATACCTCT | 4286bp |
| PED-8L | CACAACCGAATGCTGTTGACA |  |
| PED-9U | GGGCTAGCTTCCAGGTCAACT | 1714bp |
| PED-9L | GCACACCCACATCATCGTAG |  |
| PED-10U | TGTCACCGGTTGTGTAATAGC | 583bp |
| PED-10L | CTTACAGCCGTTAAAAAGC |  |
| PED-11U | CTAGCGGACTCTTACGAGATTACATATA | 633bp |
| PED-11L | GTGTATCCACATCAACACCGTCAGGTCT |  |
