# Supplementary material for: Newly Characterized Porcine Epidemic Diarrhea Virus GII Subtype Strain
Source: Transbound Emerg Dis. 2023 May 9;2023:5544724. doi: 10.1155/2023/5544724 (PMC12017209; doi:10.1155/2023/5544724)
Supplement: Supplementary Materials — Supplementary Table 1: information about samples collected in this study. Supplementary Table 2: primer sequences for S and N gene amplification. Supplementary Table 3: primer sequences for PEDV genome amplification. Supplementary Table 4: 425 PEDV strains with whole genome sequences in this study. Supplementary Table 5: 86 PEDV reference strains with complete S gene sequences in this study. Supplementary Table 6: 290 PEDV strains of the GII-a subtype with the full-length S gene sequences in this study. Supplementary Table 7: 12 representative strains for recombinant analysis. Supplementary Table 8: analysis of polarity and charge changes of the mutant aa. Supplementary Table S1: 125 reference strains used for sequence alignment and 23 strains isolated in this study. [file 5544724.f1.zip › Supplementary Table 4 (2).docx]

**Supplementary Table 4. 425 PEDV strains with whole genome sequences in this study.**

| **GenBank/Name** | **Time** | **Country** | **Classification** |
| --- | --- | --- | --- |
| KM609213.1/WS/Austria/2014 | 2014 | Austria | GII-a |
| KM609212.1/LYG/China/2014 | 2014 | China | GII-a |
| KM609211.1/PEDV-LS/China/2014 | 2014 | China | GII-a |
| JN547228.1/CHS/Beigium/1986 | 1986 | Beigium | GI-b |
| JQ023161.1/DR13/Beigium/2009 | 2009 | South Korea | GI-b |
| AF353511.1/CV777/Canada/1978 | 1978 | Canada | GI-a |
| EF185992.1/ LZC/Canada/2006 | 2006 | Canada | GI-a |
| KM189367.2/ON-018/Canada/2014 | 2014 | Canada | GII-c |
| KR265831.1/Quebec334/Canada/2014 | 2014 | Canada | GII-c |
| AF353511.1/CV777/Beigium/1978 | 1978 | Beigium | GI-a |
| KT323979.1/CV777/China/1998 | 1998 | China | GI-b |
| KC210146.1/ JS2008/China/2008 | 2008 | China | GI-b |
| KJ158152.1/ AH-M/China/2011 | 2011 | China | GI-b |
| JX524137.1/ZJCZ4/China/2011 | 2011 | China | GII-b |
| JN825712.1/BJ/China/2011 | 2011 | China | GII-a |
| MN037494.1/WHLL/China/2011 | 2011 | China | GII-a |
| KM609207.1/PEDV_14/China/2011 | 2011 | China | GII-a |
| JQ282909.1/CHFJND-3/China/2011 | 2011 | China | GII-a |
| MH726399.1/GDS42/China/2011 | 2011 | China | GII-b |
| MH726400.1/GDS45/China/2011 | 2011 | China | GII-b |
| MH726401.1/GDS41/China/2011 | 2011 | China | GII-b |
| MH726398.1/GDS37/China/2011 | 2011 | China | GII-b |
| JX188454.1/AJ1102/China/2011 | 2011 | China | GII-b |
| JX489155.1/LC/China/2011 | 2011 | China | GII-b |
| MH726393.1/GDS14/China/2011 | 2011 | China | GII-b |
| JX261936.1/CHGD_01/China/2011 | 2011 | China | GII-b |
| MK288006.1/FJzz1/China/2011 | 2011 | China | GII-a |
| MH726387.1/GDS12/China/2011 | 2011 | China | GII-b |
| MH726385.1/GDS08/China/2011 | 2011 | China | GII-b |
| MH726386.1/GDS16/China/2011 | 2011 | China | GII-b |
| MH726388.1/GDS36/China/2011 | 2011 | China | GII-b |
| MK584552.1/AJ1102/China/2011 | 2011 | China | GII-b |
| MH726390.1/GDS40/China/2011 | 2011 | China | GII-b |
| JX560761.1/ SD-M/China/2012 | 2012 | China | GI-b |
| KC189944.1/Attenuated PEDV vaccine/China/2012 | 2012 | China | GI-b |
| KC109141.1/ JS2008-2/China/2012 | 2012 | China | GI-b |
| MH726367.1/GDS26/China/2012 | 2012 | China | GII-a |
| KC140102.1/CHFJZZ-9/China/2012 | 2012 | China | GII-a |
| MH726366.1/GDS24/China/2012 | 2012 | China | GII-a |
| MG837011.1/CHNSH/China/2012 | 2012 | China | GII-a |
| KJ777677.1/FGE/China/2012 | 2012 | China | GII-a |
| KJ777678.1/ CPGEN/China/2012 | 2012 | China | GII-a |
| MH726368.1/GDS22/China/2012 | 2012 | China | GII-a |
| MH726363.1/GDS20/China/2012 | 2012 | China | GII-a |
| KF840537.1/CHZJCX-1/China/2012 | 2012 | China | GII-a |
| MH726362.1/GDS05/China/2012 | 2012 | China | GII-a |
| KM609208.1/PEDV_15F/China/2012 | 2012 | China | GII-a |
| MH726364.1/GDS17/China/2012 | 2012 | China | GII-a |
| MH107322.1/GDS23/China/2012 | 2012 | China | GII-a |
| KM609206.1/PEDV_10F/China/2012 | 2012 | China | GII-a |
| MH726397.1/GDS38/China/2012 | 2012 | China | GII-b |
| KF384500.1/CHGDGZ/China/2012 | 2012 | China | GII-b |
| MH708895.1/FJ2011/China/2012 | 2012 | China | GII-b |
| JX112709.1/GD-A/China/2012 | 2012 | China | GII-b |
| MK392335.1/LWL/China/2012 | 2012 | China | GII-b |
| KU646831.1/AH2012/China/2012 | 2012 | China | GII-b |
| MH726389.1/GDS39/China/2012 | 2012 | China | GII-b |
| MH726391.1/GDS15/China/2012 | 2012 | China | GII-b |
| KM609203.1/PEDV-1C/China/2012 | 2012 | China | GII-a |
| KU664503.1/ ZJUG/China/2013 | 2013 | China | GI-a |
| KM887144.1/CHM2013/China/2013 | 2013 | China | GI-a |
| KX839246.1/Jlu-85/China/2013 | 2013 | China | GI-a |
| KF761675.1/CHYNKM-8/China/2013 | 2013 | China | GII-b |
| KT021227.1/YN1/China/2013 | 2013 | China | GII-b |
| MH910099.1/JS-2013/China/2013 | 2013 | China | GII-a |
| KM609209.1/PEDV_CHZ/China/2013 | 2013 | China | GII-a |
| MG546687.1/CHBJ/China/2013 | 2013 | China | GII-a |
| MH726365.1/GDS25/China/2013 | 2013 | China | GII-a |
| KJ020932.1/ CHYJ/China/2013 | 2013 | China | GII-a |
| SHdt3/China | 2021 | China | GII-a |
| MH726395.1/GDS32/China/2013 | 2013 | China | GII-b |
| MH726396.1/GDS35/China/2013 | 2013 | China | GII-b |
| KT021228.1/YN15/China/2013 | 2013 | China | GII-b |
| MH726392.1/GDS44/China/2013 | 2013 | China | GII-b |
| KP765609.1/FL/China/2013 | 2013 | China | GII-b |
| MH107321.1/GDS10/China/2013 | 2013 | China | GII-c |
| KP162057.1/SC/China/2014 | 2014 | China | GI-b |
| KP728470.1/ SQ/China/2014 | 2014 | China | GI-b |
| MH726408.1/GDS09/China/2014 | 2014 | China | GI-b |
| MH726374.1/GDS11/China/2014 | 2014 | China | GII-a |
| MH726375.1/GDS19/China/2014 | 2014 | China | GII-a |
| MK841494.1/SH/China/2014 | 2014 | China | GII-a |
| MK606368.1/CH-HB1-2018/China/2014 | 2014 | China | GII-a |
| KT941120.1/HUA_14PED96/Viet Nam/2014 | 2014 | Viet Nam | GII-a |
| KM609205.1/PEDV_8C/China/2014 | 2014 | China | GII-a |
| KM242131.1/CHGDZQ/China/2014 | 2014 | China | GII-a |
| MH726370.1/GDS07/China/2014 | 2014 | China | GII-a |
| MH726369.1/GDS30/China/2014 | 2014 | China | GII-a |
| MH726376.1/GDS18/China/2014 | 2014 | China | GII-a |
| KR153326.1/CH/GDZHDM/1401/China/ | 2014 | China | GII-a |
| KX058032.1/CHJX01P5 /China/2014 | 2014 | China | GII-a |
| MH726371.1/GDS21/China/2014 | 2014 | China | GII-a |
| KT021232.1/YN144/China/2014 | 2014 | China | GII-b |
| KT021233.1/YN200/China/2014 | 2014 | China | GII-b |
| KT021231.1/YN90/China/2014 | 2014 | China | GII-b |
| KT021230.1/YN60/China/2014 | 2014 | China | GII-b |
| MH056657.1/JSX2014/ATT/China/2014 | 2014 | China | GII-c |
| KX064280.1/SD2014/China/2014 | 2014 | China | GII-a |
| MH726378.1/GDS34/China/2014 | 2014 | China | GII-a |
| MH726379.1/GDS33/China/2014 | 2014 | China | GII-a |
| MH726377.1/GDS29/China/2014 | 2014 | China | GII-a |
| KM609210.1/PEDV-LY/China/2014 | 2014 | China | GII-a |
| KY420075.1/PEDV-SX/China/2015 | 2015 | China | GI-b |
| KP403802.1/HLJBY/China/2015 | 2015 | China | GI-b |
| KX534205.1/JSLS-1/China/2015 | 2015 | China | GI-b |
| KX534206.1/JS-2/China/2015 | 2015 | China | GI-b |
| KX839251.1/mutant5/China/2015 | 2015 | China | GI-a |
| KX839247.1/mutant1/China/2015 | 2015 | China | GI-a |
| KY486713.1/A40/China/2015 | 2015 | China | GI-a |
| KY486714.1/C40/China/2015 | 2015 | China | GI-a |
| MH726381.1/GDS43/China/2015 | 2015 | China | GII-a |
| KU380331.1/ CH-SD01/China/2015 | 2015 | China | GII-c |
| KY929405.1/PT-P5/China/2015 | 2015 | China | GII-c |
| KY649107.1/CH-HNKF-16/2016 | 2016 | China | GII-a |
| MH726382.1/ GDS47/China/2016 | 2016 | China | GII-a |
| MG546690.1/CHBJ1/China/2016 | 2016 | China | GII-a |
| MF346935.1/CHJLDH/China/2016 | 2016 | China | GII-a |
| MK690502.1/ HM2017/China/2016 | 2016 | China | GII-a |
| KY928065.1/CH_hubei/China/2016 | 2016 | China | GII-a |
| MH726405.1/GDS48/China/2016 | 2016 | China | GII-a |
| MH726402.1/GDS46/China/2016 | 2016 | China | GII-a |
| KY929406.1/PT-P96/China/2016 | 2016 | China | GII-c |
| MN486588.1/Ah2016f2/China/2016 | 2016 | China | GI-b |
| MH117940.1/SDSX16/China/2016 | 2016 | China | GII-a |
| MK409657.1/ ZJ15XS0101-P16/China/2016 | 2016 | China | GII-c |
| MK409658.1/ ZJ15XS0101-P35/China/2016 | 2016 | China | GII-a |
| MK409659.1/ ZJ15XS0101-P120/China/2016 | 2016 | China | GII-a |
| MK644604.1/N7-GD/China/2017 | 2017 | China | GII-b |
| MH061338.1/CHSCZY/China/2017 | 2017 | China | GII-a |
| MH061340.1/CHSCZY/China/2017 | 2017 | China | GII-a |
| MF375374.1/CH/JXJA/China/2017 | 2017 | China | GII-a |
| MF807951.1/C3-HB/China/2017 | 2017 | China | GII-a |
| MH061337.1/CHSCZG/China/2017 | 2017 | China | GII-a |
| MK862249.1/HeN170821/China/2017 | 2017 | China | GII-a |
| MH726383.1/GDS50/China/2017 | 2017 | China | GII-a |
| MH061339.1/CHSCAZ10/China/2017 | 2017 | China | GII-a |
| MH726403.1/GDS51/China/2017 | 2017 | China | GII-a |
| MH726404.1/GDS53/China/2017 | 2017 | China | GII-a |
| MH581489.1/CHHBTS/China/2017 | 2017 | China | GII-a |
| MH708243.1/ H11-SD/China/2017 | 2017 | China | GII-a |
| MK644603.1/M3-SX2017/China/2017 | 2017 | China | GII-a |
| MH726406.1/GDS49/China/2017 | 2017 | China | GII-a |
| MH726407.1/GDS52/China/2017 | 2017 | China | GII-a |
| MK644601.1/G2-HE/China/2017 | 2017 | China | GII-a |
| MH748550.1/JS-A/China/2017 | 2017 | China | GII-b |
| SD1/China/2021 | 2021 | China | GII-a |
| ZJ3/China/2021 | 2021 | China | GII-a |
| MH061336.1/CHSCGA/China/2017 | 2017 | China | GII-a |
| MN315264.1/ AH-2018-HF1/China/2018 | 2018 | China | GI-b |
| MH061342.1/CHSCZJ/China/2018 | 2018 | China | GII-a |
| MK796238.1/CNLiaoning/China/2018 | 2018 | China | GI-b |
| MN594506.1/SX-WH/China/2018 | 2018 | China | GII-a |
| MN114121.1/CT P10/China/2018 | 2018 | China | GII-a |
| MH061343.1/CHSCMY/China/2018 | 2018 | China | GII-a |
| MK606369.1/ CH-HB2-2018/China/2018 | 2018 | China | GII-a |
| MK702008.1/ SNJ-P/China/2018 | 2018 | China | GII-a |
| MK140814.1/CH/TP-4-4/China/2018 | 2018 | China | GII-a |
| MK140811.1/CHTP-2-2/China/2018 | 2018 | China | GII-a |
| MK140812.1/CH/TP-3-1/China/2018 | 2018 | China | GII-a |
| MK140813.1/CH/TP-4-3/China/2018 | 2018 | China | GII-a |
| MK250953.1/ZJZX/China/2018 | 2018 | China | GII-a |
| MH061341.1/CHSCLS/China/2018 | 2018 | China | GII-a |
| MK138516.1/V7-HB2018/China/2018 | 2018 | China | GII-a |
| MK644605.1/ T10-HB/China/2018 | 2018 | China | GII-a |
| MG983755.1/GDgh/China/2018 | 2018 | China | GII-a |
| MK673545.1/Yunlin550/China/2018 | 2018 | China | GII-c |
| MN759311.1/GD-XL/China/2019 | 2019 | China | GII-b |
| MN056942.1/FR2019001/France/2019 | 2019 | France | GII-a |
| LT897799.1/PEDV_GER_L/Germany/1978 | 1978 | Germany | GI-b |
| LT906582.1/ Br1/Germany/1987 | 1987 | Germany | GI-b |
| MH593900.1/S236/Hungary/2018 | 2018 | Hungary | GII-a |
| KJ662670.1/KNU-1305/Korean/2013 | 2013 | Korean | GII-c |
| KJ645708.1/MEX/104/Mexico/2013 | 2013 | Mexico | GII-c |
| MH004415.1/PEDV/MEX/JAL/Mexico/2014 | 2014 | Mexico | GII-c |
| KR265766.1/Mexico329/Mexico/2014 | 2014 | Mexico | GII-c |
| MH004412.1/PEDVMEXGTO02/Mexico/2016 | 2016 | Mexico | GII-c |
| MH004414.1/PEDV/MEX/JAL/19/Mexico/2017 | 2017 | Mexico | GII-c |
| MZ268115.1/25364/2/Poland/2015 | 2015 | Poland | GII-a |
| MZ325487.1/44176/1/Poland/2016 | 2016 | Poland | GII-a |
| MZ325486.1/Poland206/Poland/2016 | 2016 | Poland | GII-a |
| MZ313556.1/0100/4T/Poland/2017 | 2017 | Poland | GII-a |
| MZ325484.1/0100/1L/Poland/2017 | 2017 | Poland | GII-a |
| MZ325485.1/0100/2M/Poland/2017 | 2017 | Poland | GII-a |
| GU937797.1/ SM98/South Korea | 2010 | South Korea | GI-a |
| JQ023162.1/DR13/South Korea/2013 | 2013 | South Korea | GI-b |
| MG781192.1/PPC 14/South Korea/2014 | 2014 | South Korea | GI-b |
| KR873435.1/KNU-141112-P10/South Korea/2014 | 2014 | South Korea | GII-c |
| KJ623926.1/K14JB01/South Korea/2014 | 2014 | South Korea | GII-c |
| MH052682.1/KNU-1703/South Korea/2017 | 2017 | South Korea | GII-c |
| MF281416.1/NB1/South Korea/2017 | 2017 | South Korea | GII-c |
| MH052681.1/KNU-1702/South Korea/2017 | 2017 | South Korea | GII-c |
| MN692784.1/PEDV-1931-1/Spain/2017 | 2017 | Spain | GII-a |
| MN692792.1/PEDV-H3-Barcelona-Vic/Spain/2019 | 2019 | Spain | GII-a |
| KP403954.1/Poltava01/Ukraine/2014 | 2014 | Ukraine | GII-c |
| KX066126.1/BJ/China/2011 | 2011 | China | GII-a |
| KM609204.1/PEDV_7C/China/2011 | 2011 | China | GII-b |
| JX647847.1/GD_1/China/2011 | 2011 | China | GII-b |
| KM089829.1/GDS01/China/2012 | 2012 | China | GII-b |
| KR818832.1/XY2013/China/2013 | 2013 | China | GII-b |
| KR818833.1/ JSHA/China/2013 | 2013 | China | GII-a |
| KX580953.1/KB/China/2013 | 2013 | China | GII-a |
| KU558701.1/ZJUG2/China/2013 | 2013 | China | GII-a |
| KJ960180.1/unknow/Viet Nam/2013 | 2013 | Viet Nam | GII-b |
| KJ960178.1/unknow/Viet Nam/2013 | 2013 | Viet Nam | GII-b |
| KJ645679.1/Minnesota86/USA/2013 | 2013 | USA | GII-c |
| LC063820.1/IBR-1/JPN/2013 | 2013 | Japan | GII-a |
| LC063821.1/IBR-2/JPN/2013 | 2013 | Japan | GII-a |
| LC063814.1/KGS-1/JPN/2013 | 2013 | Japan | GII-a |
| LC063815.1/KGS-2/JPN/2013 | 2013 | Japan | GII-a |
| KM975735.1/NC/USA/2013 | 2013 | USA | GII-c |
| LC063836.1/OKN-1/JPN/2013 | 2013 | Japan | GII-a |
| KF650373.1/ISU13-22038-IA-homogenate/USA/2013 | 2013 | USA | GII-c |
| KJ645697.1/Texas128/USA/2013 | 2013 | USA | GII-c |
| KJ778616.1/NPL-PEDv/2013/P10/USA/2013 | 2013 | USA | GII-c |
| KU558702.1/CO/P14/IC/USA/2013 | 2013 | USA | GII-c |
| KF267450.1/USA019349/USA/2013 | 2013 | USA | GII-c |
| KX683006.1/PC22A/USA/2013 | 2013 | USA | GII-c |
| KM392226.1/TC_PC168-P2/USA/2013 | 2013 | USA | GII-c |
| KF468753.1/IA1/USA/2013 | 2013 | USA | GII-c |
| KJ778615.1/NPL-PEDv/2013/USA/2013 | 2013 | USA | GII-c |
| KF650370.1/ISU13-19338E-IN-homogenate/USA/2013 | 2013 | USA | GII-c |
| KJ645688.1/Iowa96/USA/2013 | 2013 | USA | GII-c |
| KF272920.1/Colorado/USA/2013 | 2013 | USA | GII-c |
| KF452323.1/Indiana17846/USA/2013 | 2013 | USA | GII-c |
| KR078299.1/PC21A/USA/2013 | 2013 | USA | GII-c |
| KF468752.1/MN/USA/2013 | 2013 | USA | GII-c |
| KJ645683.1/NorthCarolina91/USA/2013 | 2013 | USA | GII-c |
| KF468754.1/IA2/USA/2013 | 2013 | USA | GII-c |
| KJ645685.1/Missouri93/USA/2013 | 2013 | USA | GII-c |
| KJ645693.1/Missouri102/USA/2013 | 2013 | USA | GII-c |
| LC063846.1/MYZ_1JPN/2013 | 2013 | Japan | GII-c |
| KJ645655.1/Minnesota58/USA/2013 | 2013 | USA | GII-c |
| KM975738.1/IA/USA/2013 | 2013 | USA | GII-a |
| KM975739.1/IA/2013/20849/USA/2013 | 2013 | USA | GII-a |
| KM975740.1/IL/USA/2013 | 2013 | USA | GII-c |
| KJ645695.1/Iowa106/USA/2013 | 2013 | USA | GII-c |
| KJ645696.1/Iowa107/USA/2013 | 2013 | USA | GII-c |
| KM392232.1/TC_Iowa106/USA/2013 | 2013 | USA | GII-c |
| KU975389.1/CHSCCD/China/2014 | 2014 | China | GII-a |
| KT323980.1/LNCT2/China/2014 | 2014 | China | GII-a |
| KU252649.1/YC2014/China/2014 | 2014 | China | GII-a |
| KX791060.1/CHSD/China/2014 | 2014 | China | GII-b |
| KP641662.1/OH8593-14/USA/2014 | 2014 | USA | GII-c |
| KP641663.1/OH9097-14/USA/2014 | 2014 | USA | GII-c |
| KR265824.1/USAOklahoma471/USA/2014 | 2014 | USA | GII-c |
| KR265845.1/Oklahoma466/USA/2014 | 2014 | USA | GII-c |
| KR265814.1/Kansas166/USA/2014 | 2014 | USA | GII-c |
| KR265787.1/SouthDakota285/USA/2014 | 2014 | USA | GII-c |
| LC063838.1/MYG_1JPN/2014 | 2014 | Japan | GII-a |
| KR265811.1/SouthDakota336/USA/2014 | 2014 | USA | GII-c |
| LC063837.1/AOM-2/JPN/2014 | 2014 | Japan | GII-a |
| KJ645701.1/Kansas125/USA/2014 | 2014 | USA | GII-c |
| LC063818.1/KMM-2/JPN/2014 | 2014 | Japan | GII-a |
| LC063817.1/KMM-1/JPN/2014 | 2014 | Japan | GII-a |
| LC063816.1/OKN-2/JPN/2014 | 2014 | Japan | GII-a |
| KP641661.1/OH10123-14/USA/2014 | 2014 | USA | GII-c |
| LC022792.1/Tottori2JPN/2014 | 2014 | Japan | GII-a |
| LC063828.1/TTR-2/JPN/2014 | 2014 | Japan | GII-a |
| KR265821.1/Missouri164/USA/2014 | 2014 | USA | GII-c |
| KR265819.1/Kansas431/USA/2014 | 2014 | USA | GII-c |
| KR265820.1/Missouri337/USA/2014 | 2014 | USA | GII-c |
| KR265779.1/Missouri177/USA/2014 | 2014 | USA | GII-c |
| KR265790.1/Texas424/USA/2014 | 2014 | USA | GII-c |
| KR265797.1/Kansas280/USA/2014 | 2014 | USA | GII-c |
| KR265801.1/Indiana254/USA/2014 | 2014 | USA | GII-c |
| LC063825.1/HRS-1/JPN/2014 | 2014 | Japan | GII-a |
| LC063824.1/KGW-1/JPN/2014 | 2014 | Japan | GII-a |
| LC063826.1/KGS-4/JPN/2014 | 2014 | Japan | GII-a |
| KR265774.1/SouthDakota371/USA/2014 | 2014 | USA | GII-c |
| KJ645699.1/Ohio123/USA/2014 | 2014 | USA | GII-c |
| KR265776.1/Minnesota250/USA/2014 | 2014 | USA | GII-c |
| KR265777.1/Kansas275/USA/2014 | 2014 | USA | GII-c |
| KR265780.1/Ohio343/USA/2014 | 2014 | USA | GII-c |
| KR265805.1/Iowa161/USA/2014 | 2014 | USA | GII-c |
| KR265808.1/Iowa162/USA/2014 | 2014 | USA | GII-c |
| KR265827.1/Iowa303/USA/2014 | 2014 | USA | GII-c |
| KR265810.1/Oklahoma320/USA/2014 | 2014 | USA | GII-c |
| KR265825.1/Minnesota200/USA/2014 | 2014 | USA | GII-c |
| KJ408801.1/OH1414/USA/2014 | 2014 | USA | GII-c |
| LC063810.1/AOM-1/JPN/USA/2014 | 2014 | USA | GII-c |
| KR873431.1/KNU-141112-feces/USA/2014 | 2014 | USA | GII-c |
| LC063841.1/IBR-3/JPN/Japan/2014 | 2014 | Japan | GII-a |
| LC063842.1/IBR-4/JPN/Japan/2014 | 2014 | Japan | GII-a |
| LC063840.1/NIG-2/JPN/Japan/2014 | 2014 | Japan | GII-a |
| LC063843.1/IBR-8/JPN/Japan/2014 | 2014 | Japan | GII-a |
| LC063823.1/GNM-1/JPN/Japan/2014 | 2014 | Japan | GII-a |
| LC063839.1/IWT-2/JPN/Japan/2014 | 2014 | Japan | GII-a |
| LC063813.1/IWT-4/JPN/Japan/2014 | 2014 | Japan | GII-a |
| KR265817.1/Illinois256/USA/2014 | 2014 | USA | GII-c |
| LC063811.1/FKO-1/JPN/Japan/2014 | 2014 | Japan | GII-a |
| LC063834.1/IWT-1/JPN/Japan/2014 | 2014 | Japan | GII-a |
| LC063835.1/IWT-3/JPN/Japan/2014 | 2014 | Japan | GII-a |
| KR265840.1/Ohio249/USA/2014 | 2014 | USA | GII-c |
| LC063831.1/IBR-6/JPN/Japan/2014 | 2014 | Japan | GII-a |
| LC063832.1/IBR-7/JPN/Japan/2014 | 2014 | Japan | GII-a |
| KR265781.1/Ohio295/USA/2014 | 2014 | USA | GII-c |
| KR265846.1/Missouri270/USA/2014 | 2014 | USA | GII-c |
| KR265791.1/Kentucky291/USA/2014 | 2014 | USA | GII-c |
| LC063819.1/IBR-5/JPN/Japan/2014 | 2014 | Japan | GII-a |
| LC063827.1/MIE-1/JPN/Japan/2014 | 2014 | Japan | GII-a |
| KR265772.1/Kentucky248/USA/2014 | 2014 | USA | GII-c |
| LC063829.1/GNM-2/JPN/2014 | 2014 | Japan | GII-a |
| KR265834.1/Texas435/USA/2014 | 2014 | USA | GII-c |
| KU569509.1/COLCundinamarca/USA/2014 | 2014 | USA | GII-c |
| KR265812.1/Colorado420/USA/2014 | 2014 | USA | GII-c |
| KR265792.1/Oklahoma418/USA/2014 | 2014 | USA | GII-c |
| LC063830.1/NIG-1/JPN/2014 | 2014 | Japan | GII-a |
| KR265830.1/Ohio300/USA/2014 | 2014 | USA | GII-c |
| KJ645700.1/MEX/124/Mexico/2014 | 2014 | Mexico | GII-c |
| LC063812.1/EHM-1/JPN/2014 | 2014 | Japan | GII-a |
| LC063833.1/AOM-3/JPN/2014 | 2014 | Japan | GII-a |
| KR265844.1/Missouri373/USA/2014 | 2014 | USA | GII-c |
| KJ645698.1/Ohio120/USA/2014 | 2014 | USA | GII-c |
| KR265807.1/Michigan189/USA/2014 | 2014 | USA | GII-c |
| LC063822.1/TTR-1/JPN/2014 | 2014 | Japan | GII-a |
| KR265822.1/Michigan252/USA/2014 | 2014 | USA | GII-c |
| KR265823.1/Colorado203/USA/2014 | 2014 | USA | GII-c |
| KR265803.1/Nebraska288/USA/2014 | 2014 | USA | GII-c |
| KR265784.1/Nebraska348/USA/2014 | 2014 | USA | GII-c |
| KR265809.1/Nebraska266/USA/2014 | 2014 | USA | GII-c |
| KR265767.1/Kansas432/USA/2014 | 2014 | USA | GII-c |
| KR265800.1/Indiana195/USA/2014 | 2014 | USA | GII-c |
| LT898410.1/PEDVGERL00908_K16/Germany/2014 | 2014 | Germany | GII-c |
| LT898446.1/PEDVGERL00933_K22/Germany/2014 | 2014 | Germany | GII-c |
| LT898427.1/PEDVGERL00927_K20/Germany/2014 | 2014 | Germany | GII-c |
| LT898440.1/PEDVGERL00931_K22/Germany/2014 | 2014 | Germany | GII-c |
| LT898431.1/PEDVGERL00918_K17/Germany/2014 | 2014 | Germany | GII-c |
| LT900500.1/PEDVGERL00799_K11/Germany/2014 | 2014 | Germany | GII-c |
| LT898421.1/PEDVGERL00919_K17/Germany/2014 | 2014 | Germany | GII-c |
| KR011756.1/FR001/Francey/2014 | 2014 | France | GII-a |
| LT898426.1/PEDVGERL00857_K14/Germany/2014 | 2014 | Germany | GII-c |
| LT900501.1/PEDVGERL00928_K20/ Germany/2014 | 2014 | Germany | GII-c |
| LT898415.1/PEDVGERL00855_K14/Germany/2014 | 2014 | Germany | GII-c |
| LT898438.1/PEDVGERL00932_K22/Germany/2014 | 2014 | Germany | GII-c |
| LT898447.1/PEDVGERL00862/Germany/2014 | 2014 | Germany | GII-c |
| KX883635.1/WHZHC/China/2014 | 2014 | China | GII-a |
| LM645057.1/GERL00721/Germany/2014 | 2014 | Germany | GII-a |
| LM645058.1/GERL00719/Germany/2014 | 2014 | Germany | GII-a |
| LT898445.1/PEDVGERL00798_K11/Germany/2014 | 2014 | Germany | GII-c |
| KP688354.1/Hawaii/39249/USA/2014 | 2014 | USA | GII-c |
| KR265761.1/USAHawaii/USA/2014 | 2014 | USA | GII-c |
| KJ645702.1/Ohio126/USA/2014 | 2014 | USA | GII-c |
| KT860508.1/IL20697/USA/2014 | 2014 | USA | GII-c |
| LC063844.1/KCH-1/JPN/2014 | 2014 | Japan | GII-a |
| LC063845.1/KCH-2/JPN/2014 | 2014 | Japan | GII-a |
| KM403155.1/KNU-1406-1/South Korea/2014 | 2014 | South Korea | GII-c |
| KJ399978.1/OH851/USA/2014 | 2014 | USA | GII-c |
| KM975741.1/MO/USA/2014 | 2014 | USA | GII-c |
| KY019624.1/SLOreBAS_2/Slovenia/2015 | 2015 | Slovenia | GII-a |
| KY007139.1/PEDV_Hjms/China/2015 | 2015 | China | GII-a |
| MF782686.1/NW17/China/2015 | 2015 | China | GII-a |
| KR809885.1/ CHHNAY/China/2015 | 2015 | China | GII-a |
| KY793536.1/CHGX2015750A/China/2015 | 2015 | China | GII-a |
| KT199103.1/ CHHNLH/China/2015 | 2015 | China | GII-a |
| KU982978.1/PEDVUSANebraska108/USA/2015 | 2015 | USA | GII-c |
| KU982967.1/PEDVUSAKansas126/USA/2015 | 2015 | USA | GII-c |
| KU982968.1/PEDVUSAOklahoma133/USA/2015 | 2015 | USA | GII-c |
| KR265762.1/Oklahoma1/USA/2015 | 2015 | USA | GII-c |
| KU982975.1/Missouri130/USA/2015 | 2015 | USA | GII-c |
| KU982976.1/Texas128/USA/2015 | 2015 | USA | GII-c |
| KU982977.1/Texas132/USA/2015 | 2015 | USA | GII-c |
| KU893869.1/PC22A-P95-13/USA/2015 | 2015 | USA | GII-c |
| KU893870.1/PC22A-P100-C4/USA/2015 | 2015 | USA | GII-c |
| KU893871.1/PC22A-P100-C6/USA/2015 | 2015 | USA | GII-c |
| KU893872.1/PC22A-P120/USA/2015 | 2015 | USA | GII-c |
| KU893873.1/PC22A-P160/USA/2015 | 2015 | USA | GII-c |
| KX580958.1/PC22A-P140/USA/2015 | 2015 | USA | GII-c |
| KU982969.1/Iowa127/USA/2015 | 2015 | USA | GII-c |
| KU982970.1/NorthDakota93/USA/2015 | 2015 | USA | GII-c |
| KU982966.1/Texas134/USA/2015 | 2015 | USA | GII-c |
| KU982971.1/Minnesota4/USA/2015 | 2015 | USA | GII-c |
| MF782687.1/NW8/China/2015 | 2015 | China | GII-a |
| KY019623.1/SLOreBAS-1/Slovenia/2015 | 2015 | Slovenia | GII-a |
| LT898423.1/PEDVGERL01012_K01/Germany/2015 | 2015 | Germany | GII-c |
| LT898432.1/PEDVGERL01011_K01/Germany/2015 | 2015 | Germany | GII-c |
| LT898443.1/PEDVGERL01015_K01/Germany/2015 | 2015 | Germany | GII-c |
| LT898414.1/PEDVGERL01060_K07/Germany/2015 | 2015 | Germany | GII-c |
| LT898425.1/PEDVGERL00998_K06/Germany/2015 | 2015 | Germany | GII-c |
| LT898444.1/PEDVGERL01420_K06/Germany/2015 | 2015 | Germany | GII-c |
| LT898408.1/PEDVGERL01013_K01/Germany/2015 | 2015 | Germany | GII-c |
| LT898420.1/PEDVGERL01014_K01/Germany/2015 | 2015 | Germany | GII-c |
| LT898409.1/PEDVGERL00999_K06/Germany/2015 | 2015 | Germany | GII-c |
| KU297956.1/ SLOJH_112015/Slovenia/2015 | 2015 | Slovenia | GII-a |
| LT898411.1/PEDVGERL01018_K01/Germany/2015 | 2015 | Germany | GII-c |
| LT900499.1/PEDVGERL01061_K07/Germany/2015 | 2015 | Germany | GII-c |
| LT898435.1/PEDV_Romania_L01330-K25/Romania/2015 | 2015 | Romania | GII-a |
| LT898436.1/PEDVRomaniaL01329_K25/Romania/2015 | 2015 | Romania | GII-a |
| LT898418.1/PEDVAustriaL01065_M10/Austria/2015 | 2015 | Austria | GII-a |
| LT898413.1/ PEDVGERL01020_K01/Germany/2015 | 2015 | Germany | GII-c |
| LT898433.1/PEDVAustriaL01063_M10/Austria/2015 | 2015 | Austria | GII-a |
| LT900502.1/PEDVAustriaL01062_M10/Austria/2015 | 2015 | Austria | GII-a |
| KR003452.1/15V010BEL/China/2015 | 2015 | Belgium | GII-a |
| KY007140.1/PEDV_Lnsy/China/2015 | 2015 | China | GII-a |
| KX550281.1/ZJ15XS0101_P1/China/2015 | 2015 | China | GII-a |
| KU847996.1/ZL29/China/2015 | 2015 | China | GII-a |
| MF462814.1/CHSXYL/China/2016 | 2016 | China | GII-a |
| KX812524.1/XM2_4/China/2016 | 2016 | China | GII-a |
| KY963963.1/KNU-1601/South Korea/2016 | 2016 | South Korea | GII-c |
| KY825242.1/KNU_141112_S_DEL5/South Korea/2016 | 2016 | South Korea | GII-c |
| KY825243.1/KNU-141112-S_DEL5/ORF3/South Korea/2016 | 2016 | South Korea | GII-c |
| KY825240.1/KNU-141112-S_DEL2/South Korea/2016 | 2016 | South Korea | GII-c |
| KY825241.1/KNU-141112-S_DEL2/ORF3/South Korea/2016 | 2016 | South Korea | GII-c |
| KX289955.1/HUN/5031/Hungary/2016 | 2016 | Hungary | GII-a |
| KY111278.1/PEDV_1842/Italy/2016 | 2016 | Italy | GII-a |
| KX981440.1/CHHNZZ47/China/2016 | 2016 | China | GII-a |
| KY070587.1/JSCZ1601/China/2016 | 2016 | China | GII-a |
| KX812523.1/XM1-2/China/2016 | 2016 | China | GII-a |
| MG837058.1/PC273O/USA/2017 | 2017 | USA | GII-c |
| MG334555.1/OK10240-8/USA/2017 | 2017 | USA | GII-c |
| MG334554.1/OK10240-6/USA/2017 | 2017 | USA | GII-c |
| KY499261.1/TC-PC177/USA/2017 | 2017 | USA | GII-c |
| KM609211.1/PEDV-LS/China/2014 | 2014 | China | GII-a |
| KM052365.1/NPL-PEDv/2013/p10.1/USA/2013 | 2013 | USA | GII-c |
| KM392227.1/TC_PC170-P2/USA/2013 | 2013 | USA | GII-c |
| KM392230.1/TC_PC180-P2/USA/2013 | 2013 | USA | GII-c |
| KM392229.1/TC_PC177-P2/USA/2013 | 2013 | USA | GII-c |
| KM392228.1/TC-PC173-P2/USA/2013 | 2013 | USA | GII-c |
| KM392231.1/TC_PC182-P2/USA/2013 | 2013 | USA | GII-c |
| KJ645694.1/Iowa103/USA/2013 | 2013 | USA | GII-c |
| KJ645684.1/Missouri92/USA/2013 | 2013 | USA | GII-c |
| KJ645649.1/USA/Iowa23.57/USA/2013 | 2013 | USA | GII-c |
| KJ645635.1/Indiana12.83/USA/2013 | 2013 | USA | GII-c |
| KR610993.1/UNKNOWN/USA/2014 | 2014 | USA | GII-c |
| KJ960179.1/unknow/USA/2014 | 2014 | USA | GII-c |
| KR610994.1/UNKNOWN/USA/2014 | 2014 | USA | GII-c |
