# Supplementary material for: Newly Characterized Porcine Epidemic Diarrhea Virus GII Subtype Strain
Source: Transbound Emerg Dis. 2023 May 9;2023:5544724. doi: 10.1155/2023/5544724 (PMC12017209; doi:10.1155/2023/5544724)
Supplement: Supplementary Materials — Supplementary Table 1: information about samples collected in this study. Supplementary Table 2: primer sequences for S and N gene amplification. Supplementary Table 3: primer sequences for PEDV genome amplification. Supplementary Table 4: 425 PEDV strains with whole genome sequences in this study. Supplementary Table 5: 86 PEDV reference strains with complete S gene sequences in this study. Supplementary Table 6: 290 PEDV strains of the GII-a subtype with the full-length S gene sequences in this study. Supplementary Table 7: 12 representative strains for recombinant analysis. Supplementary Table 8: analysis of polarity and charge changes of the mutant aa. Supplementary Table S1: 125 reference strains used for sequence alignment and 23 strains isolated in this study. [file 5544724.f1.zip › Supplementary Table 5 (1).docx]

**Supplementary Table 5. 86 PEDV reference strains with complete S gene sequences in this study**

| GenBank/Name | Time | Country | Classification |
| --- | --- | --- | --- |
| AF353511.1/CV777/Canada/1978 | 1978 | Canada | GI-a |
| NC_003436.1/CV777/Belgium/unknow | 1978 | Belgium | GI-a |
| LT906582.1/ Br1/Germany/1987 | 1987 | Germany | GI-a |
| LT897799.1/PEDV_GER_L/Germany/1978 | 1978 | Germany | GI-a |
| KX839246.1/Jlu-85/China/2013 | 2013 | China | GI-a |
| KX839251.1/mutant5/China/2015 | 2015 | China | GI-a |
| KU664503.1/ ZJUG/China/2013 | 2013 | China | GI-a |
| KM887144.1/CHM2013/China/2013 | 2013 | China | GI-a |
| GU937797.1/ SM98/South Korea | 2010 | South Korea | GI-a |
| EF185992.1/ LZC/Canada/2006 | 2006 | Canada | GI-a |
| MN315264.1/ AH-2018-HF1/China/2018 | 2018 | China | GI-b |
| MH726408.1/GDS09/China/2014 | 2014 | China | GI-b |
| KY420075.1/PEDV-SX/China/2015 | 2015 | China | GI-b |
| KJ158152.1/ AH-M/China/2011 | 2011 | China | GI-b |
| KT323979.1/CV777/China/1998 | 1998 | China | GI-b |
| KP162057.1/SC/China/2014 | 2014 | China | GI-b |
| JQ023162.1/DR13/South Korea/2013 | 2013 | South Korea | GI-b |
| KP403802.1/HLJBY/China/2015 | 2015 | China | GI-b |
| KP728470.1/ SQ/China/2014 | 2014 | China | GI-b |
| JX560761.1/ SD-M/China/2012 | 2012 | China | GI-b |
| KC189944.1/Attenuated PEDV vaccine/China/2012 | 2012 | China | GI-b |
| KX534206.1/JS-2/China/2015 | 2015 | China | GI-b |
| KC210146.1/ JS2008/China/2008 | 2008 | China | GI-b |
| JQ023161.1/DR13/South Korea/2009 | 2009 | South Korea | GI-b |
| KR011756.1/FR001/France/2014 | 2014 | France | S-INDEL |
| KR265760.1/Minnesota211/USA/2014 | 2014 | USA | S-INDEL |
| KJ399978.1/OH851/USA/2014 | 2014 | USA | S-INDEL |
| KR265759.1/Minnesota187/USA/2014 | 2014 | USA | S-INDEL |
| KJ645655.1/Minnesota58/USA/2013 | 2013 | USA | S-INDEL |
| KM403155.1/KNU-1406-1/South Korea/2014 | 2014 | South Korea | S-INDEL |
| KJ645649.1/USA/Iowa23.57/USA/2013 | 2013 | USA | S-INDEL |
| KR265761.1/USA/Hawaii/USA/2014 | 2014 | USA | S-INDEL |
| KM975741.1/MO/USA/2014 | 2014 | USA | S-INDEL |
| MH052681.1/KNU-1702/South Korea/2017 | 2017 | South Korea | S-INDEL |
| KU847996.1/ZL29/China/2015 | 2015 | China | GII-a |
| MK673545.1/Yunlin550/China/2018 | 2018 | China | GII-a |
| KM089829.1/GDS01/China/2012 | 2012 | China | GII-b |
| MK584552.1/AJ1102/China/2011 | 2011 | China | GII-b |
| JX647847.1/GD_1/China/2011 | 2011 | China | GII-b |
| JX112709.1/GD-A/China/2012 | 2012 | China | GII-b |
| MH726387.1/GDS12/China/2011 | 2011 | China | GII-b |
| KU646831.1/AH2012/China/2012 | 2012 | China | GII-b |
| JX261936.1/CHGD_01/China/2011 | 2011 | China | GII-b |
| KP765609.1/FL/China/2013 | 2013 | China | GII-b |
| MH708895.1/FJ2011/China/2012 | 2012 | China | GII-b |
| MK392335.1/LWL/China/2012 | 2012 | China | GII-b |
| MH726393.1/GDS14/China/2011 | 2011 | China | GII-b |
| KT021233.1/YN200/China/2014 | 2014 | China | GII-b |
| KT021232.1/YN144/China/2014 | 2014 | China | GII-b |
| KT021231.1/YN90/China/2014 | 2014 | China | GII-b |
| KT021230.1/YN60/China/2014 | 2014 | China | GII-b |
| KT021228.1/YN15/China/2013 | 2013 | China | GII-b |
| JX489155.1/LC/China/2011 | 2011 | China | GII-b |
| JX188454.1/AJ1102/China/2011 | 2011 | China | GII-b |
| KF384500.1/CHGDGZ/China/2012 | 2012 | China | GII-b |
| MH726375.1/GDS19/China/2014 | 2014 | China | GII-b |
| JX524137.1/ZJCZ4/China/2011 | 2011 | China | GII-b |
| KC140102.1/CHFJZZ-9/China/2012 | 2012 | China | GII-b |
| MH726374.1/GDS11/China/2014 | 2014 | China | GII-b |
| KT021227.1/YN1/China/2013 | 2013 | China | GII-b |
| KF761675.1/CHYNKM-8/China/2013 | 2013 | China | GII-b |
| KM609212.1/LYG/China/2014 | 2014 | China | GII-a |
| KM609207.1/PEDV_14/China/2011 | 2011 | China | GII-a |
| KF468753.1/IA1/USA/2013 | 2013 | USA | GII-a |
| KF468754.1/IA2/USA/2013 | 2013 | USA | GII-a |
| KJ408801.1/OH1414/USA/2014 | 2014 | USA | GII-a |
| KF468752.1/MN/USA/2013 | 2013 | USA | GII-a |
| KR265831.1/Quebec334/Canada/2014 | 2014 | Canada | GII-a |
| KJ645700.1/MEX/124/Mexico/2014 | 2014 | Mexico | GII-a |
| KM392229.1/TC_PC177-P2/USA/2013 | 2013 | USA | GII-a |
| KX580958.1/PC22A-P140/USA/2015 | 2015 | USA | GII-a |
| LC022792.1/Tottori2JPN/2014 | 2014 | Japan | GII-a |
| KJ451047.1/KNU-1401/South Korea/2014 | 2014 | South Korea | GII-a |
| KR818832.1/XY2013/China/2013 | 2013 | China | GII-a |
| KM609208.1/PEDV_15F/China/2012 | 2012 | China | GII-a |
| KM609210.1/PEDV-LY/China/2014 | 2014 | China | GII-a |
| KM609209.1/PEDV_CHZ/China/2013 | 2013 | China | GII-a |
| MG983755.1/GDgh/China/2018 | 2018 | China | GII-b |
| KT941120.1/HUA_14PED96/Viet Nam/2014 | 2014 | Viet Nam | GII-b |
| KM609211.1/PEDV-LS/Austria/2014 | 2014 | China | GII-a |
| KM609205.1/PEDV_8C/China/2014 | 2014 | China | GII-a |
| KM609203.1/PEDV-1C/China/2012 | 2012 | China | GII-a |
| KM609204.1/PEDV_7C/China/2011 | 2011 | China | GII-b |
| KR095279.1/CHHNQX/China/2015 | 2015 | China | Like-S-INDEL |
| KP890336.1/CHHNYF/China/2014 | 2014 | China | Like-S-INDEL |
| NC_028806.1/Italy213/Italy/2009 | 2009 | Italy | GI-b |
| Isolate |  |  |  |
| GenBank/Name | Time | Country | Classification |
| SHxn | 2020 | China | GI-b |
| JSyj10 | 2020 | China | GII-b |
| ZJ3 | 2021 | China | GII-a |
| JSdf10 | 2020 | China | GII-a |
| JSyj5 | 2020 | China | GII-a |
| JSyj8 | 2020 | China | GII-a |
| SDyt12 | 2021 | China | GII-a |
| FJ1402 | 2020 | China | GII-a |
| FJ170524 | 2020 | China | GII-a |
| FJ1401 | 2020 | China | GII-a |
| SHdt3 | 2021 | China | GII-a |
| FJ1919 | 2020 | China | GII-a |
| FJ1410 | 2020 | China | GII-a |
| FJ1617 | 2020 | China | GII-a |
| FJ1609 | 2020 | China | GII-a |
| FJ1516 | 2020 | China | GII-a |
| FJ1207 | 2020 | China | GII-a |
| FJ1312 | 2020 | China | GII-a |
| FJ1607 | 2020 | China | GII-a |
| FJ2015 | 2020 | China | GII-a |
| SD1 | 2021 | China | GII-b |
| FJ2002 | 2020 | China | GII-a |
| FJ2006 | 2020 | China | GII-a |
| FJ1912 | 2020 | China | GII-a |
