# Supplementary material for: Newly Characterized Porcine Epidemic Diarrhea Virus GII Subtype Strain
Source: Transbound Emerg Dis. 2023 May 9;2023:5544724. doi: 10.1155/2023/5544724 (PMC12017209; doi:10.1155/2023/5544724)
Supplement: Supplementary Materials — Supplementary Table 1: information about samples collected in this study. Supplementary Table 2: primer sequences for S and N gene amplification. Supplementary Table 3: primer sequences for PEDV genome amplification. Supplementary Table 4: 425 PEDV strains with whole genome sequences in this study. Supplementary Table 5: 86 PEDV reference strains with complete S gene sequences in this study. Supplementary Table 6: 290 PEDV strains of the GII-a subtype with the full-length S gene sequences in this study. Supplementary Table 7: 12 representative strains for recombinant analysis. Supplementary Table 8: analysis of polarity and charge changes of the mutant aa. Supplementary Table S1: 125 reference strains used for sequence alignment and 23 strains isolated in this study. [file 5544724.f1.zip › Supplementary Table 6 (1).docx]

**Supplementary Table 6. 290 PEDV strains of the GII-a subtype with the full-length S gene sequences in this study.**

| GenBank/Name | Time | Classification |
| --- | --- | --- |
| AF353511.1/CV777/Beigium/1978 | 1978 | GI-a |
| KM609213.1/WS/Austria/2014 | 2014 | GII-a |
| KM609212.1/LYG/China/2014 | 2014 | GII-a |
| KM609211.1/PEDV-LS/China/2014 | 2014 | GII-a |
| KM189367.2/ON-018/Canada/2014 | 2014 | GII-a |
| KR265831.1/Quebec334/Canada/2014 | 2014 | GII-a |
| JN825712.1/BJ/China/2011 | 2011 | GII-a |
| MN037494.1/WHLL/China/2011 | 2011 | GII-a |
| KM609207.1/PEDV_14/China/2011 | 2011 | GII-a |
| JQ282909.1/CHFJND-3/China/2011 | 2011 | GII-a |
| MH726367.1/GDS26/China/2012 | 2012 | GII-a |
| MG837011.1/CHNSH/China/2012 | 2012 | GII-a |
| KJ777677.1/FGE/China/2012 | 2012 | GII-a |
| KJ777678.1/ CPGEN/China/2012 | 2012 | GII-a |
| MH726368.1/GDS22/China/2012 | 2012 | GII-a |
| MH726363.1/GDS20/China/2012 | 2012 | GII-a |
| MH726362.1/GDS05/China/2012 | 2012 | GII-a |
| KM609208.1/PEDV_15F/China/2012 | 2012 | GII-a |
| MH726364.1/GDS17/China/2012 | 2012 | GII-a |
| MH107322.1/GDS23/China/2012 | 2012 | GII-a |
| KM609206.1/PEDV_10F/China/2012 | 2012 | GII-a |
| KM609203.1/PEDV-1C/China/2012 | 2012 | GII-a |
| MH910099.1/JS-2013/China/2013 | 2013 | GII-a |
| KM609209.1/PEDV_CHZ/China/2013 | 2013 | GII-a |
| MG546687.1/CHBJ/China/2013 | 2013 | GII-a |
| MH726365.1/GDS25/China/2013 | 2013 | GII-a |
| KM609205.1/PEDV_8C/China/2014 | 2014 | GII-a |
| KM242131.1/CHGDZQ/China/2014 | 2014 | GII-a |
| MH726370.1/GDS07/China/2014 | 2014 | GII-a |
| MH726376.1/GDS18/China/2014 | 2014 | GII-a |
| KR153326.1/CH/GDZHDM/1401/China/ | 2014 | GII-a |
| MH726371.1/GDS21/China/2014 | 2014 | GII-a |
| KM609210.1/PEDV-LY/China/2014 | 2014 | GII-a |
| MH726381.1/GDS43/China/2015 | 2015 | GII-a |
| KU380331.1/ CH-SD01/China/2015 | 2015 | GII-a |
| KY929405.1/PT-P5/China/2015 | 2015 | GII-a |
| KY649107.1/CH-HNKF-16/2016 | 2016 | GII-a |
| MG546690.1/CHBJ1/China/2016 | 2016 | GII-a |
| MF346935.1/CHJLDH/China/2016 | 2016 | GII-a |
| MK690502.1/ HM2017/China/2016 | 2016 | GII-a |
| KY929406.1/PT-P96/China/2016 | 2016 | GII-a |
| MH061340.1/CHSCZY/China/2017 | 2017 | GII-a |
| MF807951.1/C3-HB/China/2017 | 2017 | GII-a |
| MH061337.1/CHSCZG/China/2017 | 2017 | GII-a |
| MK862249.1/HeN170821/China/2017 | 2017 | GII-a |
| MH061339.1/CHSCAZ10/China/2017 | 2017 | GII-a |
| MK644601.1/G2-HE/China/2017 | 2017 | GII-a |
| MH061342.1/CHSCZJ/China/2018 | 2018 | GII-a |
| MK606369.1/ CH-HB2-2018/China/2018 | 2018 | GII-a |
| MK702008.1/ SNJ-P/China/2018 | 2018 | GII-a |
| MK140814.1/CH/TP-4-4/China/2018 | 2018 | GII-a |
| MH061341.1/CHSCLS/China/2018 | 2018 | GII-a |
| MK644605.1/ T10-HB/China/2018 | 2018 | GII-a |
| KJ662670.1/KNU-1305/Korean/2013 | 2013 | GII-a |
| KR873435.1/KNU-141112-P10/South Korea/2014 | 2014 | GII-a |
| KJ623926.1/K14JB01/South Korea/2014 | 2014 | GII-a |
| KP403954.1/Poltava01/Ukraine/2014 | 2014 | GII-a |
| KU558701.1/ZJUG2/China/2013 | 2013 | GII-a |
| KJ645679.1/Minnesota86/USA/2013 | 2013 | GII-a |
| KJ645686.1/Minnesota94/USA/2013 | 2013 | GII-a |
| LC063820.1/IBR-1/JPN/2013 | 2013 | GII-a |
| LC063821.1/IBR-2/JPN/2013 | 2013 | GII-a |
| LC063814.1/KGS-1/JPN/2013 | 2013 | GII-a |
| LC063815.1/KGS-2/JPN/2013 | 2013 | GII-a |
| KJ645691.1/Minnesota100/USA/2013 | 2013 | GII-a |
| KJ645706.1/Minnesota71/USA/2013 | 2013 | GII-a |
| KJ645707.1/Minnesota84/USA/2013 | 2013 | GII-a |
| KM975735.1/NC/USA/2013 | 2013 | GII-a |
| KJ645690.1/Illinois98/USA/2013 | 2013 | GII-a |
| KJ645692.1/Missouri101/USA/2013 | 2013 | GII-a |
| LC063836.1/OKN-1/JPN/2013 | 2013 | GII-a |
| KF650373.1/ISU13-22038-IA-homogenate/USA/2013 | 2013 | GII-a |
| KJ778616.1/NPL-PEDv/2013/P10/USA/2013 | 2013 | GII-a |
| KM052365.1/NPL-PEDv/2013/p10.1/USA/2013 | 2013 | GII-a |
| KF267450.1/USA019349/USA/2013 | 2013 | GII-a |
| KU893861.1/PC22A-P3/USA/2013 | 2013 | GII-a |
| KM392227.1/TC_PC170-P2/USA/2013 | 2013 | GII-a |
| KM392226.1/TC_PC168-P2/USA/2013 | 2013 | GII-a |
| KM392230.1/TC_PC180-P2/USA/2013 | 2013 | GII-a |
| KM392229.1/TC_PC177-P2/USA/2013 | 2013 | GII-a |
| KM392228.1/TC-PC173-P2/USA/2013 | 2013 | GII-a |
| KM392231.1/TC_PC182-P2/USA/2013 | 2013 | GII-a |
| KF468753.1/IA1/USA/2013 | 2013 | GII-a |
| KJ645681.1/Minnesota89/USA/2013 | 2013 | GII-a |
| KJ778615.1/NPL-PEDv/2013/USA/2013 | 2013 | GII-a |
| KF650370.1/ISU13-19338E-IN-homogenate/USA/2013 | 2013 | GII-a |
| KJ645688.1/Iowa96/USA/2013 | 2013 | GII-a |
| KJ645682.1/Minnesota90/USA/2013 | 2013 | GII-a |
| KJ645694.1/Iowa103/USA/2013 | 2013 | GII-a |
| KF272920.1/Colorado/USA/2013 | 2013 | GII-a |
| KF452323.1/Indiana17846/USA/2013 | 2013 | GII-a |
| KJ645705.1/Minnesota61/USA/2013 | 2013 | GII-a |
| KR078299.1/PC21A/USA/2013 | 2013 | GII-a |
| KM392225.1/PE103(PC21A)-P4/USA/2013 | 2013 | GII-a |
| KF468752.1/MN/USA/2013 | 2013 | GII-a |
| KJ645683.1/NorthCarolina91/USA/2013 | 2013 | GII-a |
| KJ645687.1/Minnesota95/USA/2013 | 2013 | GII-a |
| KF468754.1/IA2/USA/2013 | 2013 | GII-a |
| KJ645680.1/Illinois87/USA/2013 | 2013 | GII-a |
| KJ645689.1/Illinois97/USA/2013 | 2013 | GII-a |
| KJ645685.1/Missouri93/USA/2013 | 2013 | GII-a |
| KJ645693.1/Missouri102/USA/2013 | 2013 | GII-a |
| KJ645684.1/Missouri92/USA/2013 | 2013 | GII-a |
| KU975389.1/CHSCCD/China/2014 | 2014 | GII-a |
| KT323980.1/LNCT2/China/2014 | 2014 | GII-a |
| KU252649.1/YC2014/China/2014 | 2014 | GII-a |
| KR265824.1/USAOklahoma471/USA/2014 | 2014 | GII-a |
| KR265845.1/Oklahoma466/USA/2014 | 2014 | GII-a |
| KR265814.1/Kansas166/USA/2014 | 2014 | GII-a |
| KR265771.1/Minnesota402/USA/2014 | 2014 | GII-a |
| KR265787.1/SouthDakota285/USA/2014 | 2014 | GII-a |
| KR265816.1/Minnesota202/USA/2014 | 2014 | GII-a |
| KR265794.1/Minnesota236/USA/2014 | 2014 | GII-a |
| LC063838.1/MYG_1JPN/2014 | 2014 | GII-a |
| KR265829.1/Minnesota309/USA/2014 | 2014 | GII-a |
| KR265832.1/Minnesota379/USA/2014 | 2014 | GII-a |
| KR265811.1/SouthDakota336/USA/2014 | 2014 | GII-a |
| KR265783.1/Minnesota269/USA/2014 | 2014 | GII-a |
| KR265813.1/Minnesota271/USA/2014 | 2014 | GII-a |
| LC063837.1/AOM-2/JPN/2014 | 2014 | GII-a |
| KR265764.1/Minnesota163/USA/2014 | 2014 | GII-a |
| KJ645701.1/Kansas125/USA/2014 | 2014 | GII-a |
| KM077139.1/Minnesota188/USA/2014 | 2014 | GII-a |
| LC063818.1/KMM-2/JPN/2014 | 2014 | GII-a |
| LC063817.1/KMM-1/JPN/2014 | 2014 | GII-a |
| LC063816.1/OKN-2/JPN/2014 | 2014 | GII-a |
| KP641661.1/OH10123-14/USA/2014 | 2014 | GII-a |
| KR265769.1/Illinois197/USA/2014 | 2014 | GII-a |
| KR265821.1/Missouri164/USA/2014 | 2014 | GII-a |
| KR265819.1/Kansas431/USA/2014 | 2014 | GII-a |
| KR265789.1/Illinois307/USA/2014 | 2014 | GII-a |
| KR265820.1/Missouri337/USA/2014 | 2014 | GII-a |
| KR265782.1/Illinois308/USA/2014 | 2014 | GII-a |
| KR265779.1/Missouri177/USA/2014 | 2014 | GII-a |
| KR265797.1/Kansas280/USA/2014 | 2014 | GII-a |
| KR265801.1/Indiana254/USA/2014 | 2014 | GII-a |
| LC063825.1/HRS-1/JPN/2014 | 2014 | GII-a |
| LC063824.1/KGW-1/JPN/2014 | 2014 | GII-a |
| LC063826.1/KGS-4/JPN/2014 | 2014 | GII-a |
| KR265774.1/SouthDakota371/USA/2014 | 2014 | GII-a |
| KR265768.1/Minnesota179/USA/2014 | 2014 | GII-a |
| KJ645699.1/Ohio123/USA/2014 | 2014 | GII-a |
| KR265775.1/Minnesota338/USA/2014 | 2014 | GII-a |
| KR265776.1/Minnesota250/USA/2014 | 2014 | GII-a |
| KR265777.1/Kansas275/USA/2014 | 2014 | GII-a |
| KR265780.1/Ohio343/USA/2014 | 2014 | GII-a |
| KR265805.1/Iowa161/USA/2014 | 2014 | GII-a |
| KR265806.1/Minnesota312/USA/2014 | 2014 | GII-a |
| KR265808.1/Iowa162/USA/2014 | 2014 | GII-a |
| KR265785.1/Illinois259/USA/2014 | 2014 | GII-a |
| KR265827.1/Iowa303/USA/2014 | 2014 | GII-a |
| KR265810.1/Oklahoma320/USA/2014 | 2014 | GII-a |
| KR265825.1/Minnesota200/USA/2014 | 2014 | GII-a |
| KR265788.1/Minnesota306/USA/2014 | 2014 | GII-a |
| KJ408801.1/OH1414/USA/2014 | 2014 | GII-a |
| KR265770.1/Illinois255/USA/2014 | 2014 | GII-a |
| LC063810.1/AOM-1/JPN/USA/2014 | 2014 | GII-a |
| KR873431.1/KNU-141112-feces/USA/2014 | 2014 | GII-a |
| LC063841.1/IBR-3/JPN/Japan/2014 | 2014 | GII-a |
| LC063842.1/IBR-4/JPN/Japan/2014 | 2014 | GII-a |
| LC063840.1/NIG-2/JPN/Japan/2014 | 2014 | GII-a |
| LC063843.1/IBR-8/JPN/Japan/2014 | 2014 | GII-a |
| KR265773.1/Minnesota281/USA/2014 | 2014 | GII-a |
| LC063823.1/GNM-1/JPN/Japan/2014 | 2014 | GII-a |
| LC063839.1/IWT-2/JPN/Japan/2014 | 2014 | GII-a |
| LC063813.1/IWT-4/JPN/Japan/2014 | 2014 | GII-a |
| KR265817.1/Illinois256/USA/2014 | 2014 | GII-a |
| LC063811.1/FKO-1/JPN/Japan/2014 | 2014 | GII-a |
| LC063834.1/IWT-1/JPN/Japan/2014 | 2014 | GII-a |
| LC063835.1/IWT-3/JPN/Japan/2014 | 2014 | GII-a |
| KR265841.1/Minnesota408/USA/2014 | 2014 | GII-a |
| KR265842.1/Minnesota409/USA/2014 | 2014 | GII-a |
| KR265840.1/Ohio249/USA/2014 | 2014 | GII-a |
| LC063831.1/IBR-6/JPN/Japan/2014 | 2014 | GII-a |
| LC063832.1/IBR-7/JPN/Japan/2014 | 2014 | GII-a |
| KR265818.1/Minnesota290/USA/2014 | 2014 | GII-a |
| KR265781.1/Ohio295/USA/2014 | 2014 | GII-a |
| KR265846.1/Missouri270/USA/2014 | 2014 | GII-a |
| KR265791.1/Kentucky291/USA/2014 | 2014 | GII-a |
| LC063819.1/IBR-5/JPN/Japan/2014 | 2014 | GII-a |
| KR265804.1/Illinois333/USA/2014 | 2014 | GII-a |
| LC063827.1/MIE-1/JPN/Japan/2014 | 2014 | GII-a |
| KR265772.1/Kentucky248/USA/2014 | 2014 | GII-a |
| LC063829.1/GNM-2/JPN/2014 | 2014 | GII-a |
| KU569509.1/COLCundinamarca/USA/2014 | 2014 | GII-a |
| KR265812.1/Colorado420/USA/2014 | 2014 | GII-a |
| KR265792.1/Oklahoma418/USA/2014 | 2014 | GII-a |
| LC063830.1/NIG-1/JPN/2014 | 2014 | GII-a |
| KR265830.1/Ohio300/USA/2014 | 2014 | GII-a |
| LC063812.1/EHM-1/JPN/2014 | 2014 | GII-a |
| LC063833.1/AOM-3/JPN/2014 | 2014 | GII-a |
| KR265844.1/Missouri373/USA/2014 | 2014 | GII-a |
| KR265802.1/Illinois260/USA/2014 | 2014 | GII-a |
| KR265786.1/Illinois261/USA/2014 | 2014 | GII-a |
| KR265815.1/Illinois194/USA/2014 | 2014 | GII-a |
| KR265828.1/Minnesota265/USA/2014 | 2014 | GII-a |
| KR265778.1/Minnesota276/USA/2014 | 2014 | GII-a |
| KR265799.1/Minnesota159/USA/2014 | 2014 | GII-a |
| KJ645698.1/Ohio120/USA/2014 | 2014 | GII-a |
| KR265807.1/Michigan189/USA/2014 | 2014 | GII-a |
| KR265795.1/Illinois201/USA/2014 | 2014 | GII-a |
| KR265796.1/Illinois262/USA/2014 | 2014 | GII-a |
| LC063822.1/TTR-1/JPN/2014 | 2014 | GII-a |
| KR265763.1/Illinois176/USA/2014 | 2014 | GII-a |
| KR265822.1/Michigan252/USA/2014 | 2014 | GII-a |
| KR265823.1/Colorado203/USA/2014 | 2014 | GII-a |
| KR265826.1/Illinois258/USA/2014 | 2014 | GII-a |
| KR265803.1/Nebraska288/USA/2014 | 2014 | GII-a |
| KR265765.1/Nebraska287/USA/2014 | 2014 | GII-a |
| KR265784.1/Nebraska348/USA/2014 | 2014 | GII-a |
| KR265809.1/Nebraska266/USA/2014 | 2014 | GII-a |
| KR265767.1/Kansas432/USA/2014 | 2014 | GII-a |
| KR265800.1/Indiana195/USA/2014 | 2014 | GII-a |
| KR265793.1/Minnesota212/USA/2014 | 2014 | GII-a |
| KR265843.1/Minnesota372/USA/2014 | 2014 | GII-a |
| KY007139.1/PEDV_Hjms/China/2015 | 2015 | GII-a |
| MF782686.1/NW17/China/2015 | 2015 | GII-a |
| KR809885.1/ CHHNAY/China/2015 | 2015 | GII-a |
| KT199103.1/ CHHNLH/China/2015 | 2015 | GII-a |
| KU982979.1/PEDVUSAMinnesota131/USA/2015 | 2015 | GII-a |
| KU982981.1/PEDV/USA/Minnesota124/USA/2015 | 2015 | GII-a |
| KU982967.1/PEDVUSAKansas126/USA/2015 | 2015 | GII-a |
| KU982968.1/PEDVUSAOklahoma133/USA/2015 | 2015 | GII-a |
| KU982974.1/Minnesota123/USA/2015 | 2015 | GII-a |
| KR265762.1/Oklahoma1/USA/2015 | 2015 | GII-a |
| KU982975.1/Missouri130/USA/2015 | 2015 | GII-a |
| KU893869.1/PC22A-P95-13/USA/2015 | 2015 | GII-a |
| KU893870.1/PC22A-P100-C4/USA/2015 | 2015 | GII-a |
| KU893871.1/PC22A-P100-C6/USA/2015 | 2015 | GII-a |
| KU893872.1/PC22A-P120/USA/2015 | 2015 | GII-a |
| KU893873.1/PC22A-P160/USA/2015 | 2015 | GII-a |
| KU982969.1/Iowa127/USA/2015 | 2015 | GII-a |
| KU982970.1/NorthDakota93/USA/2015 | 2015 | GII-a |
| KU982973.1/Minnesota135/USA/2015 | 2015 | GII-a |
| KU982972.1/Minnesota129/USA/2015 | 2015 | GII-a |
| KU982971.1/Minnesota4/USA/2015 | 2015 | GII-a |
| KY007140.1/PEDV_Lnsy/China/2015 | 2015 | GII-a |
| KY963963.1/KNU-1601/South Korea/2016 | 2016 | GII-a |
| KY070587.1/JSCZ1601/China/2016 | 2016 | GII-a |
| MG334554.1/OK10240-6/USA/2017 | 2017 | GII-a |
| KY499261.1/TC-PC177/USA/2017 | 2017 | GII-a |
| KM609213.1/WS/Austria/2014 | 2014 | GII-a |
| KC140102.1/CHFJZZ-9/China/2012 | 2012 | GII-a |
| MH726366.1/GDS24/China/2012 | 2012 | GII-a |
| KF840537.1/CHZJCX-1/China/2012 | 2012 | GII-a |
| KJ020932.1/ CHYJ/China/2013 | 2013 | GII-a |
| MH726374.1/GDS11/China/2014 | 2014 | GII-a |
| MH726375.1/GDS19/China/2014 | 2014 | GII-a |
| KT941120.1/HUA_14PED96/Viet Nam/2014 | 2014 | GII-a |
| MH726369.1/GDS30/China/2014 | 2014 | GII-a |
| KX058032.1/CHJX01P5 /China/2014 | 2014 | GII-a |
| MH056657.1/JSX2014/ATT/China/2014 | 2014 | GII-a |
| KX064280.1/SD2014/China/2014 | 2014 | GII-a |
| MH726378.1/GDS34/China/2014 | 2014 | GII-a |
| MH726379.1/GDS33/China/2014 | 2014 | GII-a |
| MH726377.1/GDS29/China/2014 | 2014 | GII-a |
| MH726382.1/ GDS47/China/2016 | 2016 | GII-a |
| KY928065.1/CH_hubei/China/2016 | 2016 | GII-a |
| MH726405.1/GDS48/China/2016 | 2016 | GII-a |
| MH726402.1/GDS46/China/2016 | 2016 | GII-a |
| MH117940.1/SDSX16/China/2016 | 2016 | GII-a |
| MH061338.1/CHSCZY/China/2017 | 2017 | GII-a |
| MF375374.1/CH/JXJA/China/2017 | 2017 | GII-a |
| MH726383.1/GDS50/China/2017 | 2017 | GII-a |
| MH726403.1/GDS51/China/2017 | 2017 | GII-a |
| MH726404.1/GDS53/China/2017 | 2017 | GII-a |
| MH581489.1/CHHBTS/China/2017 | 2017 | GII-a |
| MH708243.1/ H11-SD/China/2017 | 2017 | GII-a |
| MK644603.1/M3-SX2017/China/2017 | 2017 | GII-a |
| MH726406.1/GDS49/China/2017 | 2017 | GII-a |
| MH726407.1/GDS52/China/2017 | 2017 | GII-a |
| MH061336.1/CHSCGA/China/2017 | 2017 | GII-a |
| MN594506.1/SX-WH/China/2018 | 2018 | GII-a |
| MH061343.1/CHSCMY/China/2018 | 2018 | GII-a |
| MK140811.1/CHTP-2-2/China/2018 | 2018 | GII-a |
| MK140812.1/CH/TP-3-1/China/2018 | 2018 | GII-a |
| MK140813.1/CH/TP-4-3/China/2018 | 2018 | GII-a |
| MK250953.1/ZJZX/China/2018 | 2018 | GII-a |
| MK138516.1/V7-HB2018/China/2018 | 2018 | GII-a |
| MG983755.1/GDgh/China/2018 | 2018 | GII-a |
| MK673545.1/Yunlin550/China/2018 | 2018 | GII-a |
| KY793536.1/CHGX2015750A/China/2015 | 2015 | GII-a |
| KU982980.1/Minnesota125/USA/2015 | 2015 | GII-a |
| KU982976.1/Texas128/USA/2015 | 2015 | GII-a |
| KU982977.1/Texas132/USA/2015 | 2015 | GII-a |
| KX550281.1/ZJ15XS0101_P1/China/2015 | 2015 | GII-a |
| MF462814.1/CHSXYL/China/2016 | 2016 | GII-a |
| KX812524.1/XM2_4/China/2016 | 2016 | GII-a |
| KX981440.1/CHHNZZ47/China/2016 | 2016 | GII-a |
| KX812523.1/XM1-2/China/2016 | 2016 | GII-a |
