# Supplementary material for: Newly Characterized Porcine Epidemic Diarrhea Virus GII Subtype Strain
Source: Transbound Emerg Dis. 2023 May 9;2023:5544724. doi: 10.1155/2023/5544724 (PMC12017209; doi:10.1155/2023/5544724)
Supplement: Supplementary Materials — Supplementary Table 1: information about samples collected in this study. Supplementary Table 2: primer sequences for S and N gene amplification. Supplementary Table 3: primer sequences for PEDV genome amplification. Supplementary Table 4: 425 PEDV strains with whole genome sequences in this study. Supplementary Table 5: 86 PEDV reference strains with complete S gene sequences in this study. Supplementary Table 6: 290 PEDV strains of the GII-a subtype with the full-length S gene sequences in this study. Supplementary Table 7: 12 representative strains for recombinant analysis. Supplementary Table 8: analysis of polarity and charge changes of the mutant aa. Supplementary Table S1: 125 reference strains used for sequence alignment and 23 strains isolated in this study. [file 5544724.f1.zip › Supplementary Table 7 (1).docx]

**Supplementary Table 7. 12 representative strains for recombinant analysis.**

| Number | Year | Strain name | GenBank |
| --- | --- | --- | --- |
| 1 | 1978 | CV777 | AF353511.1 |
| 2 | 2014 | SQ | KP728470.1 |
| 3 | 2008 | JS2008 | KC210146.1 |
| 4 | 2009 | DR13 | JQ023161.1 |
| 5 | 2013 | Minnesota58 | KJ645655.1 |
| 6 | 2014 | KNU-1406-1 | KM403155.1 |
| 7 | 2014 | FR001 | KR011756.1 |
| 8 | 2014 | OH851 | KJ399978.1 |
| 9 | 2011 | PEDV-7C | KM609204.1 |
| 10 | 2013 | FL2013 | KP765609.1 |
| 11 | 2011 | AJ1102 | JX188454.1 |
| 12 | 2013 | YN1 | KT021227.1 |
