# Supplementary material for: Newly Characterized Porcine Epidemic Diarrhea Virus GII Subtype Strain
Source: Transbound Emerg Dis. 2023 May 9;2023:5544724. doi: 10.1155/2023/5544724 (PMC12017209; doi:10.1155/2023/5544724)
Supplement: Supplementary Materials — Supplementary Table 1: information about samples collected in this study. Supplementary Table 2: primer sequences for S and N gene amplification. Supplementary Table 3: primer sequences for PEDV genome amplification. Supplementary Table 4: 425 PEDV strains with whole genome sequences in this study. Supplementary Table 5: 86 PEDV reference strains with complete S gene sequences in this study. Supplementary Table 6: 290 PEDV strains of the GII-a subtype with the full-length S gene sequences in this study. Supplementary Table 7: 12 representative strains for recombinant analysis. Supplementary Table 8: analysis of polarity and charge changes of the mutant aa. Supplementary Table S1: 125 reference strains used for sequence alignment and 23 strains isolated in this study. [file 5544724.f1.zip › Supplementary Table 8 (1).docx]

**Supplementary Table 8. Analysis of polarity and charge changes of the mutant aa.**

| (None,None) to (polar,negative/positive-charge) |
| --- |
| P15S |
| S54I |
| M55G |
| N56E |
| G67A |
| I70H |
| E71P |
| I119T |
| N130I |
| D162- |
| A178S |
| R202G |
| T236I |
| S247P |
| D129S |
| S458A |
| A609E |
| S1048A |
| (None,None) to (polar/non-polar,None) |
| I5T |
| -58Q |
| -59G |
| -60V |
| -61N |
| –140N |
| I163- |
|  |
| (None,None) to (None,negative/positive-charge) |
| Y83H |
| D85R |
| Q88H |
| Y156H |
| R158S |
| G160H |
| K161S |
| R200S |
| T210E |
| E229Q |
| H302Q |
| D314Q |
| I360T |
| E369Q |
| N711D |
| G1177D |
| S1236R |
| R1306Q |
