# Supplementary material for: Newly Characterized Porcine Epidemic Diarrhea Virus GII Subtype Strain
Source: Transbound Emerg Dis. 2023 May 9;2023:5544724. doi: 10.1155/2023/5544724 (PMC12017209; doi:10.1155/2023/5544724)
Supplement: Supplementary Materials — Supplementary Table 1: information about samples collected in this study. Supplementary Table 2: primer sequences for S and N gene amplification. Supplementary Table 3: primer sequences for PEDV genome amplification. Supplementary Table 4: 425 PEDV strains with whole genome sequences in this study. Supplementary Table 5: 86 PEDV reference strains with complete S gene sequences in this study. Supplementary Table 6: 290 PEDV strains of the GII-a subtype with the full-length S gene sequences in this study. Supplementary Table 7: 12 representative strains for recombinant analysis. Supplementary Table 8: analysis of polarity and charge changes of the mutant aa. Supplementary Table S1: 125 reference strains used for sequence alignment and 23 strains isolated in this study. [file 5544724.f1.zip › Supplementary Table S1 (1).docx]

Supplementary Table S1. 125 reference strains used for sequence alignment and 23 GII genotype strains isolated in this study.

| **GI genotype reference strain** | **GenBank accession number** |
| --- | --- |
| CV777-1978 | AF353511.1 |
| **GII-a subgroup reference strain** | |
| PEDV SH-S | MK841494.1 |
| PEDV 15F | KM609208.1 |
| GDS22 | MH726368.1 |
| GDS30 | MH726369.1 |
| WHLL-S | MN037494.1 |
| CHNSH-2012-5 | MG837011.1 |
| GDS05 | MH726362.1 |
| CHBJ92015 | MG546687.1 |
| GDS17 | MH726364.1 |
| GDS25 | MH726365.1 |
| PEDV 14 | KM609207.1 |
| GDS20 | MH726363.1 |
| CHZJCX-1 | KF840537.1 |
| PEDV CHZ | KM609209.1 |
| BJ-2011 | JN825712.1 |
| LNCT2 | KT323980.1 |
| PEDV 1C | KM609203.1 |
| GDS29 | MH726377.1 |
| GDS34 | MH726378.1 |
| GDS33 | MH726379.1 |
| GDS26 | MH726367.1 |
| GDS10 | MH107321.1 |
| SDSX16 | MH117940.1 |
| JSX2014ATT | MH056657.1 |
| PT-P5 | KY929405.1 |
| PT-P96 | KY929406.1 |
| CH-HB1-2018 | MK606368.1 |
| C3-HB2017 | MF807951.1 |
| CT P10-S | MN114121.1 |
| GDS18 | MH726376.1 |
| CHGDZQ | KM242131.1 |
| GDS07 | MH726370.1 |
| CHSCZJ2018 | MH061342.1 |
| CNLiaoning252018 | MK796238.1- |
| CHSCZG2017 | MH061337.1 |
| ZJ15XS0101-P120-S | MK409659.1 |
| ZJ15XS0101-P35-S | MK409658.1 |
| ZJ15XS0101-P16-S | MK409657.1 |
| CHNSH-2016-4 | MG837012.1 |
| HLJ2015DP1-1 | KR351293.1 |
| CHFJND | KC140102.1 |
| CHYJ13 | KJ020932.1 |
| GDS23 | MH107322.1 |
| GDS13 | MH726373.1 |
| GDS21 | MH726371.1 |
| HeN170821 | MK862249.1 |
| CHHNYF14 | KP890336.1 |
| CHHNQX-314 | KR095279.1 |
| GDS24 | MH726366.1 |
| PEDV-8C | KM609205.1 |
| AH2012 | KC210145.1 |
| PEDV-WS | KM609213.1 |
| JSHA2013 | KR818833.1 |
| PEDV-LY | KM609210.1 |
| **Isolated GII-a subgroup strain** | |
| FJ170524 | OQ349217 |
| FJ1401 | OQ349205 |
| FJ1312 | OQ349204 |
| FJ1410 | OQ349207 |
| FJ1516 | OQ349208 |
| FJ1207 | OQ349203 |
| FJ1912 | OQ349212 |
| FJ1617 | OQ349211 |
| FJ1609 | OQ349210 |
| FJ2006 | OQ349215 |
| FJ1402 | OQ349206 |
| JSyj5 | OQ349219 |
| JSyj8 | OQ349220 |
| SDyt12 | OQ349223 |
| JSdf10 | OQ349218 |
| FJ1607 | OQ349209 |
| FJ2015 | OQ349216 |
| ZJ3 | OQ349226 |
| FJ2002 | OQ349214 |
| FJ919 | OQ349213 |
| SHdt3 | OQ349201 |
| **GII-b subgroup reference strain** | |
| PEDV JS-A | MH748550.1 |
| GDS44 | MH726392.1 |
| GDS39 | MH726389.1 |
| AJ1102 | JX188454.1 |
| GDS40 | MH726390.1 |
| GD-XL | MN759311.1 |
| GDS15 | MH726391.1 |
| GDS36 | MH726388.1 |
| CHGDZHDM1401 | KR153326.1 |
| LWL-S | MK392335.1 |
| GD-A | JX112709.1 |
| GDS08 | MH726385.1 |
| GD-1 | JX647847.1 |
| GDS12 | MH726387.1 |
| GDS16 | MH726386.1 |
| CHGDGZ2012 | KF384500.1 |
| GDS19 | MH726375.1 |
| GDS11 | MH726374.1 |
| GDS14 | MH726393.1 |
| LC | JX489155.1 |
| CHYNKM-8 | KF761675.1 |
| YN1 | KT021227.1 |
| YN15 | KT021228.1 |
| YN60 | KT021230.1 |
| YN90 | KT021231.1 |
| YN144 | KT021232.1 |
| YN200 | KT021233.1 |
| G2-HE2017-S | MK644601.1 |
| CH-SD01 | KU380331.1 |
| ZJCZ4 | JX524137.1 |
| CHFJZZ | KC140102.1 |
| GDS42 | MH726399.1 |
| GDS45 | MH726400.1 |
| GDS32 | MH726395.1 |
| GDS37 | MH726398.1 |
| GDS38 | MH726397.1 |
| GDS35 | MH726396.1 |
| GDS41 | MH726401.1 |
| GDS49 | MH726406.1 |
| GDS52 | MH726407.1 |
| GDS46 | MH726402.1 |
| G2-HE2017 | MK644601.1 |
| M3-SX2017 | MK644603.1 |
| FJzz1 | MK288006.1 |
| GDgh | MG983755.1 |
| CH_hubei_2016 | KY928065.1 |
| GDS51 | MH726403.1 |
| GDS53 | MH726404.1 |
| GDS43 | MH726381.1 |
| ZJZX2018-C10 | MK250953.1 |
| GDS50 | MH726383.1 |
| CH-HNKF-16 | KY649107.1 |
| PEDV 10F | KM609206.1 |
| CHJLDH2016 | MF346935.1 |
| HM2017 | MK690502.1 |
| T10-HB2018 | MK644605.1 |
| CHBJ112016 | MG546690.1 |
| CHGDZH021401 | KR153325.1 |
| CHGDZHDM1401 | KR153326.1 |
| CHSCLS2018 | MH061341.1 |
| CHJXJA2017 | MF375374.1 |
| GDS48 | MH726405.1 |
| H11-SD2017 | MH708243.1 |
| CHHBTS2017 | MH581489.1 |
| CHSCAZ102017 | MH061339.1 |
| CH-HB2-2018 | MK606369.1 |
| CHSCZY1032017 | MH061340.1 |
| SNJ-P | MK702008.1 |
| CH/TP-4-4 | MK140814.1 |
| XY2013 | KR818832.1 |
| **Isolated GII-b subgroup strain** | |
| JSyj10 | OQ349221 |
| SD1 | OQ349222 |
